# Extraction of Natural-Based Raw Materials Towards the Production of Sustainable Man-Made Organic Fibres

**DOI:** 10.3390/polym16243602

**Published:** 2024-12-23

**Authors:** Ana Catarina Vale, Liliana Leite, Vânia Pais, João Bessa, Fernando Cunha, Raul Fangueiro

**Affiliations:** 1 Fibrenamics, Institute of Innovation on Fiber-Based Materials and Composites, University of Minho, 4800-058 Guimarães, Portugal; vaniapais@fibrenamics.com (V.P.); joaobessa@fibrenamics.com (J.B.); fernandocunha@fibrenamics.com (F.C.); rfangueiro@det.uminho.pt (R.F.); 2 Centre for Textile Science and Technology (2C2T), Department of Textile Engineering, University of Minho, 4800-058 Guimarães, Portugal

**Keywords:** biomass pre-treatment, bioresources, man-made organic fibres, natural-based polymers, renewable sources, polymer extraction, sustainable materials

## Abstract

Bioresources have been gaining popularity due to their abundance, renewability, and recyclability. Nevertheless, given their diverse composition and complex hierarchical structures, these bio-based sources must be carefully processed to effectively extract valuable raw polymeric materials suitable for producing man-made organic fibres. This review will first highlight the most relevant bio-based sources, with a particular focus on promising unconventional biomass sources (terrestrial vegetables, aquatic vegetables, fungi, and insects), as well as agroforestry and industrial biowaste (food, paper/wood, and textile). For each source, typical applications and the biopolymers usually extracted will also be outlined. Furthermore, acknowledging the challenging lignocellulosic structure and composition of these sources, an overview of conventional and emerging pre-treatments and extraction methods, namely physical, chemical, physicochemical, and biological methodologies, will also be presented. Additionally, this review aims to explore the applications of the compounds obtained in the production of man-made organic fibres (MMOFs). A brief description of their evolution and their distinct properties will be described, as well as the most prominent commercial MMOFs currently available. Ultimately, this review concludes with future perspectives concerning the pursuit of greener and sustainable polymeric sources, as well as effective extraction processes. The potential and main challenges of implementing these sources in the production of alternative man-made organic fibres for diverse applications will also be highlighted.

## 1. Introduction

The extraordinary progress in the development of fibrous-based materials, such as textiles, high-performance, or multifunctional products, is irrefutably linked with the extensive exploitation of fossil-based plastics (also designated as synthetic polymers) [1]. Given these polymers’ properties, such as their light weight, durability, and versatility [2], the rate of plastic production has exponentially risen worldwide in recent decades, reaching 400.3 Mt in 2022 [3]. Moreover, due to the absence of efficient plastic waste management infrastructures, only 9% of global plastic production is recycled (mechanical and chemical) [3]. Consequently, the amount of discarded plastic has proportionally increased, either disposed of in landfills or released into the environment, where it ends up in the ocean or waterways, triggering a complex cascade of ecological drawbacks. Recent research has shown the adverse impact of rising plastic deposits in our ecosystems [4,5,6,7,8,9], essentially originating from progressive plastic fragmentation and the subsequent release of particles with different diameters, namely macro- (>20 mm diameter), meso- (5–20 mm), micro- (<5 mm), or nanoplastics (1 µm–1 nm). Additionally, this causes the leaching of additives from plastics, which have known toxic effects on living organisms [1,9]. A similar trend is observed in fibrous materials, where synthetic petroleum-based fibres dominate the market. Subsequently, there is a growing demand for eco-friendly, natural-derived polymers that contribute to carbon-neutral processes, are biodegradable, and can be utilised across various sectors, including the textile, healthcare, and protection industries [10,11,12].

There has been an acceleration of research in this promising area to address the need previously presented and to increase the cost-effectiveness of bioresource biorefineries by coupling biofuel, high-value products, and biopolymer production using waste and wastewater extracts. Such a strategy improves overall sustainability by lowering costs and carbon emissions in biorefineries, eventually contributing towards the much-touted circular, net-zero carbon future economies [13].

This review contributes to the existing literature with a comprehensive overview of alternative sources, particularly involving the exploitation of biomass and biowaste. It focuses on unconventional and more sustainable bio-based sources that could provide valuable polymers (polysaccharides, proteins, phenolic compounds, and lipids) and derived monomers (e.g., glucose, amino acids, and fatty and dicarboxylic acids) suitable for the synthesis of bio-based polymers, envisaging the production of man-made organic fibres. In this sense, this review highlights some abundant sources of biomass and biowaste that could be better explored to supply building blocks for both the production and functionalisation of sustainable man-made organic fibres. Nonetheless, to enable the efficient extraction of biopolymers and their subsequent monomers, the most relevant methodologies for bio-extraction (i.e., the extraction of biopolymers) will be briefly outlined, ranging from conventional approaches to emerging and more environmentally friendly techniques. Moreover, this review provides a discussion of the evolution of man-made fibres and their production, which, in turn, highlights the emergence of new directions to produce more sustainable fibres from greener and renewable sources. New perspectives will be afforded to embrace natural-based polymers and bio-based monomers as sustainable alternatives to fossil-derived sources, to produce valuable and multifunctional man-made organic fibres.

For the above-mentioned topics that this review paper explores, a bibliographic search was performed in the ScienceDirect, ResearchGate, and Google Scholar databases using a combination of the following keywords: biomass; biowaste; sustainable sources; renewable sources; agroforestry biowaste; industrial biowaste; valorisation; biorefinery; biopolymers; green chemistry; chemical pre-treatments; mechanical pre-treatments; physiochemical pre-treatments; biological pre-treatments; lignocellulosic extraction; sustainable fibres; man-made organic fibres; and fibre production. In this regard, it should be mentioned that a remarkable number of documents have been published in the last decade related to these different terms. Only research papers, reviews, and book chapters are considered in the comprehensive analysis presented in the following sections.

## 2. Bioresources: Biomass and Biowaste

In addressing environmental and sustainability issues, there is an urgent need to explore the potential of abundant biomass and biowaste as privileged polymeric sources available for fibre production. Both biomass and biowaste have been exploited for conversion into electricity, heat, and advanced biofuels, either through thermochemical or biochemical processes [14], but there is another sustainable route to explore for these renewable materials, which is the extraction of valuable biopolymers.

**Figure 1:**
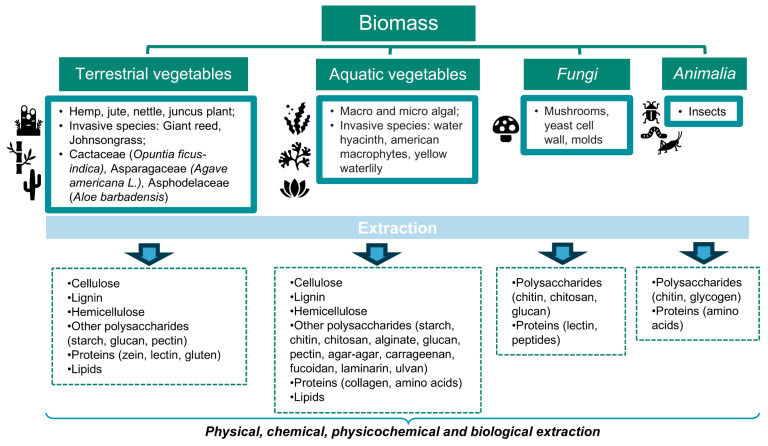
Schematic illustration of main biomass sources and resulting biopolymers extracted.

**Figure 2:**
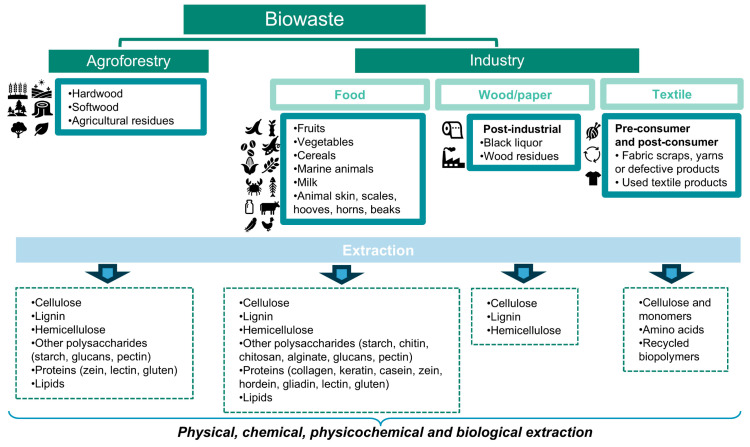
Schematic illustration of most relevant biowaste sources and resulting biopolymers extracted.

Given that distinct biomass definitions have been provided in different contexts (e.g., ecology and energy) [14,15,16,17], this review proposes the following distinction between biomass and biowaste sources. Biomass or primary biomass comprises any organic material that could be taken directly from nature and, as illustrated in Figure 1, the most abundant resources are vegetable (terrestrial and aquatic), fungal, and Animalia species. On the other hand, biowaste consists of residual biomass obtained from different biomass processing, as shown in Figure 2, particularly from agroforestry and industrial (food, paper/wood, textile) sectors. In fact, contaminated biowaste from municipal waste was excluded from this discussion, as it presents additional costs and challenges for effective biopolymer extraction.

Concerning biomass, the main sources that should be highlighted, due to their abundance and biopolymeric composition, are suggestively classified as terrestrial and aquatic vegetables, fungal, and animal species (see Figure 1).

### 2.1. Terrestrial Vegetables

Regarding terrestrial vegetables, the relevance of cotton, flax, or ramie as the plant species recurrently used to obtain valuable plant-based natural fibres is well known. However, it is also recognised the negative environmental impact attributed to their cultivation and manufacturing, particularly for cotton [10,11,12]. Hence, searching for alternative sources to obtain valuable biocompounds suitable for fibre production, there are some terrestrial vegetable species that could reduce these environmental concerns, including water use, pesticide and fertiliser application, eutrophication, and greenhouse gas emissions [18]. In this regard, the most promising terrestrial vegetable species that could be considered are hemp (*Cannabis sativa Linn.*), jute (*Corchorus capsularis*), common nettle (*Urtica dioica* L.), and Juncus plant (*Juncaceae*).

Hemp, one of the world’s oldest bast plants, is known for its exceptionally strong cellulose fibres [19,20]. Beyond its fibres, hemp biomass—including hurds, leaves, and inflorescences—offers great potential for synthesising bioproducts such as high-quality microbial protein [21] and biopolymers like polyhydroxyalkanoates (PHA) and polyhydroxylbutyrate (PHB). These bioproducts can be applied across various industries, including food, textile, and packaging [19,22]. Particularly for the recovery of both cellulosic and non-cellulosic components from hemp, some studies have highlighted extraction methodologies based on deep eutectic solvents (DES) [23,24,25,26] to produce greener functional and bioactive nanofibres with distinct applications [23,27,28]. In addition, jute (*Corchorus capsularis* L.) is another source of non-wood lignocellulosic content [29,30,31], mostly cultivated in Southeast Asian countries for the extraction of bast fibres with textile and high-quality paper pulp production applications [32]. In fact, jute has been exploited for the extraction of nanocellulose (e.g., cellulose nanofibrils, cellulose nanocrystals), which presents a unique structure and excellent mechanical properties and low thermal expansion coefficient and transparency [33], which are suitable as reinforcing fillers in biodegradable polymeric matrices [34,35,36,37]. Moreover, in comparison with other non-wood bast fibres, a typical jute fibre exhibits high lignin content [38], predominantly composed of syringyl (S) units, that enable a relatively easier delignification [39]. This high lignin content has a negative effect on fibre quality and separation [32], particularly for textile purposes, which is why jute is often subjected to a delignification process to obtain low-lignin fibres. The S-lignin removed during this process is a valuable renewable aromatic biopolymer, with the potential to replace petroleum-based aromatic polymers and to produce biochemicals and bio-based materials [40].

The common nettle (*Urtica dioica* L.) is a perennial plant with minimal pesticide and fertiliser requirements that is widely distributed all over the world. Attending the high cellulosic content in nettle leaves [41], this renewable source has a high potential for nanocellulose extraction (e.g., nanocrystalline and nanofibrillated cellulose or hairy cellulose nanocrystals) [41,42]. These nanocelluloses are described as lightweight nanofillers with high stable dispersity, offering high strength and a superior strength-to-weight ratio [41], making them suitable for a wide range of nanocomposite materials. Moreover, nettle is one of the most used medicinal plants in traditional medicine and recently has also attracted interest in modern medicine [43]. These attractive pharmacological/medicinal properties of nettle are essentially due to the peculiar phytochemical composition of the nettle plant (root, leaves, stem, flowers, seed), which is rich in terpenoids, carotenoids, fatty acids, polyphenols, essential amino acids, polysaccharides–glucans, lectins, flavonoids, vitamins, and minerals, among others [43,44]. Some studies have reported the high potential of nettle leaf extracts with valuable biological activity, including antioxidant, antibacterial, anti-inflammatory, anti-cancer, anti-diabetic, and hepato-protective properties [45,46]. In this sense, the effective extraction of these biologically active compounds and their further incorporation into fibrous materials could contribute to their application in the biomedical field.

The Juncus plant (*Juncus effusus* L.) is a perennial plant that can grow in distinct environments, particularly in wet areas. It exhibits stems with a high amount of cellulose and therefore has great potential to serve as an excellent source for producing cellulose derivatives, including micro- and nanocellulose fibres and cellulose nanocrystals [47,48,49,50]. Moreover, some studies have described the effective mechanical and structural reinforcement of these cellulose derivatives when incorporated as a filler in the development of different fibrous and nanocomposite materials [47,48,49,51].

On the other hand, some invasive species could also be considered as an alternative lignocellulosic source due to their high biomass production and growth rate. Some that can be pointed out are as follows: giant reed (*Arundo donax* L.), a perennial grass recognised by its high productivity and low requirements [52,53,54], and Johnsongrass (*Sorghum halepense* L.), another perennial plant with high tolerance and the ability to grow in different environmental conditions, as it is constituted by fibres with mechanical properties comparable to some non-wood fibres [55,56,57,58,59]. Additionally, there is an emergent trend to explore abundant cactus and succulent plants as a rich source of polysaccharides, polyphenols, and natural cellulosic fibres for textile, food, paper production, and biocomposites applications [60]. In this regard, Cactaceae species (opuntia species, such as *Opuntia maxima*, *Opuntia ficus-indica* L.) [60,61,62,63], Asparagaceae species (agaves plants, such as *Agave americana* L.) [64,65,66,67] and Asphodelaceae species (Aloe plants, such as *Aloe barbadensis*) [68,69,70] are good examples of attractive cactus and succulent plants.

### 2.2. Aquatic Vegetables

Concerning aquatic flora, macroalgal (seaweed) and microalgal species have major significance, not only for biopolymer extraction, but also for providing precursors that could be further converted into bioplastics. This class of biosources has the advantage of presenting a simple cultivation process, which can contribute to the elimination of excess nutrients from the surrounding environment and help reduce greenhouse gas emissions, freshwater consumption, and potential deforestation [71]. Moreover, attending to the extracellular composition of macroalgae, namely the high content of polysaccharides, their extraction has gained particular interest [72]. In this regard, the most valuable polysaccharides extracted are agar–agar and carrageenan, commonly obtained from red algae (*Rhodophyta*); alginates, fucoidans, and laminarin, derived from brown algae (*Phaeophyta*); and granular starch and ulvan resulting from green algae (*Chlorophyta*) [13]. Furthermore, several macroalgal species have been described as having high levels of cellulosic content. These species include the following: green macroalgae such as *Cladophora* (20–45%), *Chaetomorpha* (37–41%), *Rhizoclonium* (39%), and *Ulva* (2–19%); red macroalgae such as *Griffithsia* (22%), *Ceramium* (19%), *Chondria* (16%), *Corallina* (15%), *Gelidiella* (11–14%), *Gracilaria* (11%) and *Hypnea* (11%); and also a few brown macroalgae, such as *Laminaria* (1–20%), *Fucus* (14%), and *Halidrys* (14%) [13,73,74].

On the other hand, microalgae are unicellular organisms that grow rapidly even in polluted environments (wastewater) [71,74]. Some species, such as *Chlamydomonas* [75], *Chlorella vulgaris* [76], and *Chlorella pyrenoidosa* [77]), have been mostly exploited for biorefinery production (e.g., biodiesel, biogas, bioethanol), where the resulting by-products could be used to obtain biopolymers (mainly, cellulose and starch) and polyesters (such as polyhydroxyalkanoate, polybutylene succinate, and polylactic acid). In this regard, since the productivity of microalgae is intrinsically dependent on different factors involved in carbon fixation and photosynthesis processes (among others, light, temperature, culture density, species cultivated, and culture medium conditions), microalgae have not been extensively used for biopolymer extraction as macroalgae [13].

Numerous studies have analysed the compositional analysis of both macro- and microalgae, with some examples presented in Table 1. It is worth noting that the significant variation in polymeric content (e.g., cellulose) can largely be attributed to the influence of cultivation conditions. This was demonstrated by Aguirre and Bassi [78], who emphasised the importance of well-designed cultivation strategies to maximise cellulose production [73,78].

Some invasive aquatic species, such as water hyacinth (*Eichhornia crassipes*), Canadian waterweed (*Elodea canadensis Michx*), and yellow waterlily (*Nymphaea mexicana Zucc.*), could also be considered abundant sources of lignocellulosic content, without negative effects on global diversity, since they need to be regularly removed from waterways and the biomass obtained is generally disposed of without further use.

Water hyacinth (*Eichhornia crassipes*) is a widely available free-floating aquatic weed, notable for its high content of cellulosic fibres. It has traditionally been used as a textile raw material in the furniture and household handicraft industries, but recent studies have already reported its potential for the synthesis of cellulose nanofibres [85,89,90]. Notably, it has been reported that *Eichhornia crassipes* fibres have better properties than glass fibres, including higher tensile strength, stiffness, heat resistance, and corrosion resistance properties, and have great potential as a filler in polymer composites [85,90].

Waterweeds or American macrophytes, such as *Elodea canadensis* and *Elodea nuttallii*, have also been proliferating at a high-speed rate in waterways over the last few decades. Therefore, this aquatic biomass has been periodically removed and disposed of without further use. To repurpose them, a few works have reported their composition, revealing lignin, cellulose, and hemicellulose within the typical range for fast-growing species, along with a relatively high protein content [87]. In addition, *E. canadensis* contains bioactive compounds, including chlorophyll, carotenoids, ascorbic acid, flavonoids, and phenolic acid, which have gained interest in the biosynthesis of various kinds of nanoparticles suitable for biomedical applications [88].

Yellow waterlily (*Nymphaea mexicana Zucc.*) is another aquatic plant with the potential to grow rapidly and spread into waterways. This aquatic plant has not been substantially explored, however, some studies have performed its physicochemical characterisation, which has described different valuable bioactive extracts with anti-inflammatory and anti-microbial properties [91,92].

### 2.3. Fungi

Fungi, one of the largest groups of eukaryotes, include a diverse variety of organisms ranging from microscopic unicellular yeasts to macroscopic multicellular mushrooms, and are the second-largest community of organisms on the planet [93]. They play a major role in balancing the environment, the carbon cycle, and the mobilisation of biogenic elements, such as nitrogen and phosphorus [94]. Respecting their structural composition, fungi present similar structures to crustaceans, particularly in their polysaccharides, which are among the most significant bioactive components. Fungal polysaccharides have been extensively used in the cosmetic, food, and health product industries [95]. One of those polysaccharides is chitin, the main component of the cell walls of fungi (1–15%) [96], with fungi being the second most common source of chitin after crustaceans [97]. Moreover, fungal chitosan can also be obtained through deacetylation of the chitin [98]. The only fungal source in which chitosan naturally occurs together with chitin is the cell wall of the zygomycetes, a filamentous fungus [99,100]. Fungal chitosan has several advantages over crustacean chitosan, such as better availability and consistent quality. Hence, some research papers have proposed this alternative chitosan source for producing biocompatible materials, such as absorbable sutures [99], and sustainable textiles [100].

Besides chitin, the cell wall of fungi includes many β-glucans, which are linked with different types of β-glycosidic bonds. Since most of them have a highly branched structure and helix conformation, fungal β-glucans have demonstrated various biological functions such as immunomodulatory, antioxidation, anti-inflammatory, and antibacterial [93,101,102]. Moreover, due to the high content, quality, and availability of proteins contained in fungal organisms, attention is being paid to the fungal proteins and their potential application in food and health areas [94]. Among all fungal proteins, lectin is one of the most studied proteins due to its bioactive properties, since it can recognise and interact with a wide range of cell surface glycans/glycoproteins [103]. Several studies have been conducted with lectins derived from mushrooms (82% of fungal lectins), but there is also lectin present in moulds and yeasts, which represents around 15% and 3% of fungal lectins, respectively [94].

### 2.4. Animalia

From Animalia, insects are one of the most diverse and abundant arthropods. They have been considered a valuable food source since their exoskeleton is constituted of chitin (10–15%), proteins (30–45%), fat (25–40%), and minerals [104]. Recent research has been conducted to explore edible insects as an alternative source of chitin, chitosan [97,105], and proteins [106,107,108]. Some orders have been studied and extensively characterised, namely *Blattodea*, *Coleoptera*, *Hymenoptera*, *Lepidoptera*, and *Orthoptera* [109]. Compared to crustaceans, insects typically have a lower inorganic content, allowing extraction methods to be carried out under milder conditions [109], as previously discussed by Philibert et al. [110]. Therefore, insects present the potential to be used as an alternative source of chitin and chitosan [97,111,112,113,114].

Concerning biowaste, two main sectors will be explored within the scope of this review, namely agroforestry and industry (food, wood/paper, and textile). These could provide valuable raw materials to be explored for biopolymer extraction (see Figure 2).

### 2.5. Agroforestry Residues (Hardwood, Softwood, and Agricultural Residues)

Agroforestry consists of the integration of farming systems with woody perennials in a balanced ecological system that can improve both crop growth and soil fertility [115,116]. Hence, this new concept is rising worldwide to overcome some adverse effects of agricultural practices [117].

Subsequently, from agroforestry practices diverse by-products can be obtained, such as hardwood, softwood, and agricultural residues. These residues can yield various renewable chemicals and materials, with the lignocellulosic fraction (or lignocellulose feedstock)—comprising lignin, cellulose, hemicellulose, and tannin—being the most abundant raw material [118]. This abundance highlights the potential of utilising this biowaste for the production of high-value compounds [119]. Thorenz et al. [120] assessed the bioeconomic potential of agroforestry residues in the European Union, identifying wheat straw, maize stover, barley straw, and rape straw as the most promising agricultural sources. These crops have a high lignocellulosic concentration, exceeding 80% of their dry matter. However, their potential remains largely underutilised, with only 8% of agricultural waste currently being exploited. In forestry, coniferous species such as spruce and pine are the most notable sources, containing approximately 70% lignocellulosic content.

Distinct applications have been attributed to the residual biomass from agroforestry, such as the production of biofuels (biodiesel, bioalcohol), biogas, and bioenergy [121,122]. Along with that, there is also the possibility of using the biomass for the synthesis of chemicals, building block materials, biochar, paper and packaging, oils, bioplastics (e.g., polyhydroxyalkanoates, polylactic acid), and bioadsorbents [117].

Due to their wide availability, wood and its residues have great importance in energy production, the construction sector, and the pulp and paper industry [123,124]. In fact, wood and its inherent main constituents, carbohydrates (cellulose and hemicelluloses) and lignin, are also of great interest as sources of green chemicals, fuels, polymers, and novel nanomaterials. However, the recalcitrant nature of lignocellulose, related to lignin content, could significantly reduce the effective valorisation of this biomass. In this regard, softwood presents a more recalcitrant structure compared with hardwood [125], as presented in Table 2, which could involve more aggressive delignification processes.

Nevertheless, different bio-derived materials can be obtained from soft- and hardwood residues. The most recent advances have highlighted particular interest in emergent materials, such as nanocellulose, hemicellulose, lignin, and activated carbon [123]. For instance, nanocellulose has been applied to design advanced materials with distinct applications in the energy, electronics, and biomedical fields. Moreover, hemicellulose, a heteropolysaccharide that could present distinct compositions, structures, and concentrations [181], can be widely used in the biomedical field and the transportation packaging field. Lignin is a complex and water-insoluble amorphous polymer with phenolic moieties that, in wood, could range from 15 to 30 wt% [182] as the major byproduct from the paper industry and lignocellulosic biorefineries. This macromolecule is suitable to produce bioactive (nano) materials for biomedical purposes (e.g., drug delivery). In the end, all wood residues could be used to produce activated carbon materials, which have potential in energy storage and environmental adsorption areas [123,183].

In addition to wood residues (both soft- and hardwood), which are associated with environmental concerns such as deforestation, abundant agricultural residues—such as stalks, husk, straw, and pomace—serve as sustainable alternatives for lignocellulosic sources. These agricultural residues offer a diverse range of lignocellulosic biomass, with their composition varying significantly depending on factors such as climate, cultivation conditions, crop varieties, extraction methods, and geographical distribution [184].

### 2.6. Industrial Waste

Concerning industrial waste, the main sectors contributing to massive waste and subsequent environmental concerns are the food, wood/paper, and textile industries.

The growing global population and the corresponding demand for processed food have led to a significant accumulation of by-products from food processing industries, creating serious environmental and economic challenges [185]. Nevertheless, a variety of industrial food waste including fruits and vegetables (e.g., banana, citrus, apple, tomato, and potato skin waste, mango, grape, and pumpkin seed wastes, coffee waste, bagasse, and sugar molasses waste, and peanut husk), grains (e.g., wheat, soybean, and rice husk) or animal-derived waste (e.g., dairy, seafood, meat, and fish processing) could be applied to extraction and synthesis of biopolymers [185]. Some examples of polymers that could be obtained are polyhydroxyalkanoates (PHA), polyhydroxybutyrate (PHB), and polysaccharides, such as xanthan gum, polylactic acid (PLA), and peptides. These present themselves as promising substitutes for conventional petroleum-based polymers.

Generally, industrial fruit, vegetable, and grain waste could include distinct residues, such as skin, seed, fruit, bagasse, molasse, and husk. All of those mentioned are composed of organic matter available for extraction as follows: structural polysaccharides (cellulose, starch, hemicellulose, pectin, glucan); storage proteins (zein, lectin, hordein, gliadin); lignin (and related phenolic compounds); and lipids (and derived fatty acids) [185,186,187]. Concerning the biopolymer extraction from this complex and diverse lignocellulosic biowaste, different works reported in the literature have compiled and discussed distinct strategies for an efficient valorisation of this abundant source in biorefineries and synthesis of bio-derived polymers, for example, PLA and PHA [185,188,189,190,191,192].

Seafood waste consists of crustacean (crabs, shrimp, lobster) shells and other inedible fractions. These present a complex hierarchical organisation consisting of crystalline alpha-chitin nanofibres embedded in a protein matrix with minerals [141,193]. Hence, these exoskeletons have been extensively exploited as the main biosource for chitin and chitosan extraction. Several works have reported different approaches for the extraction and characterisation of these structural polysaccharides from crustacean waste [137,138,139,140,141].

In the meat and fish processing industries, considerable amounts of biowaste are generated and discarded in landfills or incinerated [142,143], causing severe environmental concerns. The meat by-products are mostly from pigs, chickens, and cattle [194] and might comprise hair, wool, feathers, bones, hooves, horns, and beaks. The fish by-products could include carcasses, heads, skin, scales, tails, and viscera. These residues have been considered for their potential to obtain animal-derived proteins and other valuable compounds, such as enzymes, oils, and minerals. Some research has been conducted to develop effective extraction of animal-derived proteins, such as collagen [142,144,145,146], gelatine [147,148,149], and keratin [143,150,151,152]. These biomaterials are suitable for producing multifunctional materials, for example, nanoparticles, nanofibres, scaffolds, and hydrogels with distinct applications (e.g., food, cosmetics, and biomedical fields). Moreover, based on the content of fatty acids obtained from animal fat, meat industry waste has also been considered to produce biodiesel and co-products as PHA [153,154,155].

On the other hand, the dairy industry has also increased worldwide [195], leading to an intensified generation of organic waste from milk and other processed dairy products (e.g., cheese, yoghurt, butter, and ice cream) [156,157]. Dairy waste usually consists of solid and liquid wastes and is rich in lipids and proteins. These molecules can degrade and release toxic substances, which are a danger to biodiversity [196]. Due to the environmental impact of dairy wastewater, recent biotechnological processes have been established for the treatment and appropriate valorisation of dairy by-products [156]. Among a variety of by-products, whey stands out as a notable resource that is rich in lactoferrin and casein. It has been progressively exploited for casein extraction [158,159,160,161] and for biosynthesis of xanthan gum [159,162] and polyhydroxyalkanoates [163,164].

Concerning the pulp and paper industry, the most valuable by-products generated are black liquor and woody residues. Woody residues could be processed, and their valorisation may follow a similar pathway as common lignocellulosic materials, namely bioenergy and bioproducts (composites, biofuels) production [197]. On the other hand, black liquor is the black/brown aqueous phase obtained from the pulping process, generated when the pulp (cellulosic material) is washed after the delignification step. Distinct chemical pulp extraction treatments have been applied for paper production, but the Kraft process is the dominant process. The resulting black liquor mostly constitutes lignin, polysaccharides (as hemicellulose), resinous compounds, and some soluble salt ions [165,167]. Commonly, black liquor is incinerated or gassed as a source of fuel energy. Nonetheless, this waste liquor can be used as a preferable source of lignin and hemicellulose for the synthesis of polyhydroxyalkanoates, biogas, and carboxylic acids (e.g., succinic, glycolic, lactic, formic, and acetic acids), which are essential monomers for polymer synthesis [166,168].

Alongside the industries previously described, the textile industry is undoubtedly responsible for a significant fraction of the amount of waste every year. It has been estimated that around 92 million tonnes of textile waste are produced worldwide, which is one of the biggest contributors of plastic waste [198,199]. Generally, textile waste is defined as any discarded piece of fabric or clothing that is unfit for its original purpose [199]. This can be carried out during several phases of the process as follows: production; manufacturing or processing; defective textiles (pre-consumer waste); and after their end of life, including discarded clothing, bedding, or automotive textiles (post-consumer or post-industrial waste) [169,200].

Involving pre-consumer and post-consumer textile waste, several innovations have been conducted for collecting (deployment of the Internet of Things, IoT, or the use of smart tags and sensors for traceability), sorting (technology for identification and classification of textile waste), and the recycling textiles (innovative mechanical, chemical, biochemical, or thermal recycling). These new systems are implemented to address global demands on the reuse and recycling of waste [199].

Depending on the recycling process conducted with textile waste, different valuable products could be obtained, such as regenerated fibres, fillers, biofuels, bioplastics, biochar, cellulose derivatives, and amino acids. Mechanical recycling regenerates fibres (both natural and synthetic) for applications similar or not to those of the original fabrics without using chemicals. Yet, it is hampered by the availability and quality of waste materials, which generally reduces the quality and strength of the recycled fibres originated [170]. On the other hand, chemical recycling could also be applied to synthetic fibres (such as polyester, polyamide, or acrylic), natural fibres (such as cotton, wool, or silk), or blended fabrics [171]. This process involves depolymerising waste into their oligomers or monomers (e.g., glucose, bis(2-hydroxyethyl) terephthalate, BHET, dimethyl terephthalate, ethylene glycol) through diverse methods, namely hydrolysis, glycolysis, methanolysis, or solvolysis, which frequently requires harsh chemicals. Chemical recycling consists of different steps as follows: separation; purification; and subsequent repolymerisation to produce recycled polymers [172]. In addition, through esterification and etherification processes, glucose derived from cotton waste can be transformed into useful textile additives, for example, cellulose acetate or sodium carboxymethylcellulose [173]. Biological recycling produces similar results to chemical reprocessing, using microorganisms (bacteria, fungi, and algae) and their secreted enzymes for biodegradation [171]. Nevertheless, due to the resistance of synthetic fibres, biological methods are mainly suitable for natural fibres [171]. Particularly for natural fibres, glucose, and amino acids can be recovered to apply to the synthesis of new bio-based polymers and fibres [174,175]. Furthermore, the glucose recovered from cellulosic fibres could be a low-cost raw material for bio-derived monomers manufacture, such as carboxylic acids (e.g., succinic acid) [179], by way of microbial fermentation processes. Based on PET-cotton textile waste, enzymes have been employed in recovering glucose and polyester monomers [176,177,178].

## 3. Pre-Treatments and Extraction Methods: Insights, Main Advantages, and Disadvantages

Lignocellulosic resources are the point of attention in replacing the dependence on fossil fuels, due to their availability and abundance [201]. Therefore, distinct pre-treatments and extraction methods can be conducted to accurately treat lignocellulosic biomass and biowaste from diverse sources previously described.

**Figure 3:**
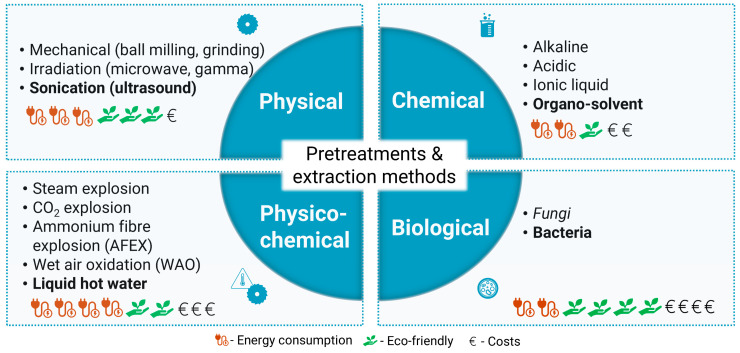
Distinct pre-treatments and extraction methods used to treat lignocellulosic biomass and biowaste from diverse sources. The pictogram under each class of treatments, namely physical, chemical, physicochemical, and biological intends to demonstrate their energy consumption, environmental, and cost issues in comparison to one another.

**Figure 4:**
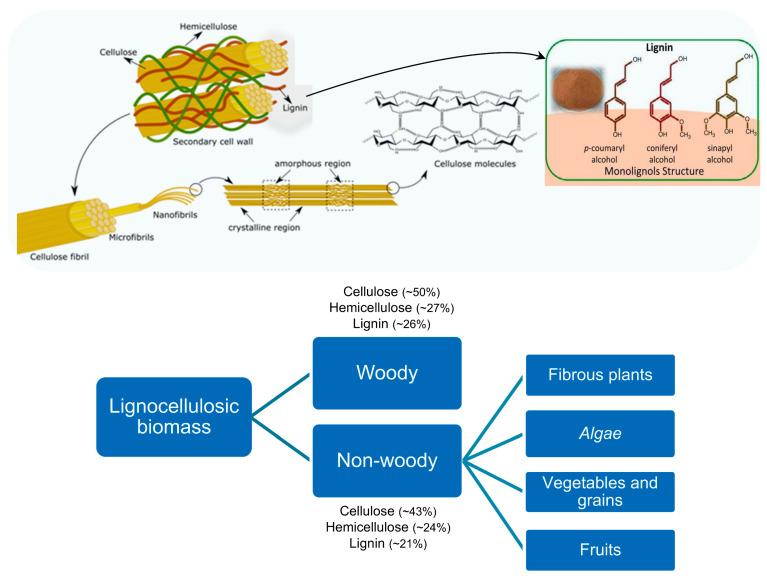
Schematically illustration of the hierarchical structure of lignocellulosic resources and their diverse sources (adapted with permission from [203], Copyright^®^ 2022, Elsevier, and [204], Copyright^®^ 2020, licensed by MDPI, Basel, Switzerland under CC-BY 4.0).

**Figure 5:**
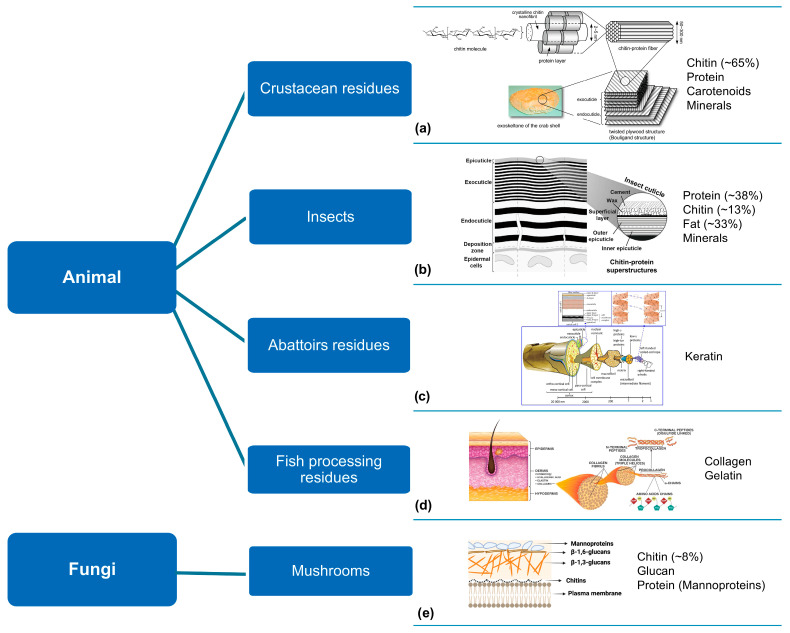
Schematically illustration of hierarchical structure of animal and fungal bioresources: (**a**) crustacean exoskeleton (reprinted with permission from [141], Copyright© 2009, American Chemical Society); (**b**) insect exoskeleton (reprinted with permission from [205], Copyright© 2022, licensed by American Chemical Society under CC-BY 4.0); (**c**) internal structure of a wool fibre and intrinsic keratin arrangement (adapted with permission from [206], Copyright© 2022, licensed by Wiley Periodics LLC on behalf of Institute of Food Technologists under CC-BY-NC-ND 4.0; (**d**) animal skin collagen (reprinted with permission from [145], Copyright© 2022, licensed by MDPI, Basel, Switzerland under CC-BY 4.0); and (**e**) fungal wall matrix (adapted with permission from [207], Copyright© 2023, licensed by MDPI, Basel, Switzerland under CC-BY 4.0).

For instance, multiple techniques can be followed to extract cellulose and its derivatives, namely physical, chemical, physicochemical, and biological, as schematically illustrated in Figure 3. Essentially, depending on the complexity of the hierarchical structure of the renewable resources schematically illustrated in Figure 4 and Figure 5, appropriate pulping technologies as well as pulping conditions should be applied. These choices significantly influence specific features and the environmental impact of the resulting materials. In this regard, Shukla et al. [201] and Padhi et al. [202] reported an extensive discussion of the main advantages and disadvantages of these different methods commonly used to manage biomasses. Hence, the following sub-sections are intended to highlight the most relevant aspects concerning these different processing and extraction approaches.

### 3.1. Physical Methods

Generally, physical methods include diverse processing technologies, comprising mechanical, microwave, or gamma irradiation and sonication processes. These methodologies can induce the structural disruption of biomass/waste, leading to size reduction and surface area increase. Physical methods are consistently used as pre-treatments of different biomass/waste since they provide crucial processing of a wide range of resources for further extractive routes. In the case of lignocellulosic resources, sonication [60,129,208,209,210,211] and irradiation [24,54,118,212,213,214,215,216] methods are commonly used to trigger the delignification process, which is essential for the effective extraction of cellulose and other valuable structural polysaccharides and proteins. Besides the simplicity and versatility of these processes, they involve the application of a wide range of temperature and pressure conditions, which implies some costs in energy consumption [201].

### 3.2. Chemical Methods

Chemical methods are undoubtedly the most commonly employed for extraction, particularly when dealing with challenging resources such as lignocellulosic biomass or waste (Figure 4), as well as animal residues and fungi biomass featuring chitin-protein or a protein-based hierarchical microstructure (Figure 5).

Among others, chemical approaches comprise the following different solvent systems: alkaline (or alkali); acidic; organo-solvent (or organosolv); ionic liquid; and deep eutectic. These can break down the complex structure exhibited by diverse biomass and biowaste, converting their valuable building blocks (e.g., polysaccharides, proteins, phenolic compounds, and lipids) to be more prone to being extracted. Particularly for lignocellulosic resources (Figure 4), these chemical methods have been recurrently used to efficiently disrupt the lignin barrier, transforming the crystalline structure of lignocellulosic mass into an amorphous form and offering an easier extraction of cellulosic content. Among different chemical methods, alkaline and acidic are the most used due to their high extraction yields; however, these conventional methods present a high environmental impact.

Overall, it is undeniable that acidic treatments are the most relevant extraction processes, especially when combined with other physical treatments, such as ultrasound. However, they present several negative aspects, including hemicellulose degradation, corrosiveness, and the formation of inhibitory species that could reduce their efficiency [217].

In this sense, alternative methods have risen. Some examples are organo-solvents [212,213,215,218,219,220,221,222,223,224,225,226], ionic liquids [27,227,228,229,230], and deep eutectic solvents [23,24,26,231], claiming to be greener solvent systems that can be easily reused or recycled, and that generally provide good extraction yields under mild operational conditions [201,232].

### 3.3. Physicochemical Methods

Based on the combination of physical and chemical methods, physiochemical processes have been developed and explored specifically at an industrial scale for processing some agroforestry and food industrial residues. Among others, the physiochemical methods frequently used are steam explosion, carbon dioxide explosion, ammonium fibre explosion (AFEX), wet air oxidation (WAO), and liquid hot water. It has been reported that these methods have provided an enhanced production of sugar along with a maximum level of delignification [201,233]. Moreover, it should be mentioned that these methodologies allow efficient extraction of hemicellulose and modified cellulose for their further processing and appropriate valorisation in biorefinery and bioenergy purposes [116,234]. Nevertheless, as physical methods, these methods require extreme temperature and pressure conditions, leading to higher energy consumption costs. Consequently, they are considered less environmentally friendly.

### 3.4. Biological Methods

As an alternative to the previously mentioned extraction processes, greener extractions could be provided by the anaerobic digestion process. These are commonly designed biological or enzymatic methods.

Currently, biological treatments could involve fungi, bacteria, microbial consortiums, and enzymes. Since commercial and pure enzymes derived from bacteria and fungi are too expensive, a cheaper solution could be provided by microbial extracellular enzymes isolated from diverse sources (e.g., plants and animals) [235,236].

Essentially, these biological approaches rely on specific enzymes, mainly hemicellulases and cellulases [237]. Nevertheless, new perspectives have risen to improve the biological extraction of lignocellulose biomass or waste. These include the search for more efficient functional strains or enzymes, additional research into the enzymatic hydrolysis characteristics and mechanisms, improving the strain’s performance through genetic engineering, and immobilising hydrolases on nanomaterials (e.g., nanoparticles, nanotubes, nanosheets), providing better thermostability and reusability of enzymes [235,236].

From this diversity, as highlighted in recent works [122,184,204,209,214,224,236,238,239,240], enzymatic procedures (or bio-pulping) stand out as the most environmentally friendly approach. These are followed by manufacturing processes, including steaming complemented by mechanical processes and soda (or alkaline) chemical procedures.

Generally, on behalf of biological processes, enzymatic processes provide higher selective degradation of lignin at low temperatures and atmospheric pressure. Nonetheless, they require long processing time and tremendous costs involved in enzyme acquisition or microbiological/fungal cultures. Hence, the combination of these biological hydrolyses with other effective methods (chemical and physicochemical) may reduce the time necessary for the whole process, which also helps to strengthen the lignocellulose dissociation efficiency [235].

## 4. Man-Made Organic Fibres

Until the early years of the previous century, natural fibres were employed as a multifunctional material for several applications; however, they still present significant challenges that must be addressed. Effectively, they have limited availability, and their culture has a high environmental impact, as well as a low production rate insufficient to meet the recent growing consumer demands [12]. To overcome the limitations described, man-made fibres have risen as promising alternatives through two main categories: regenerated fibres with natural-based compositions and synthetic fibres composed of petroleum-based synthetic polymers.

Man-made synthetic fibres are mostly composed of polyesters, polypropylene, and polyamides with high molecular weight, exhibiting high production rate, low cost, durability, and multifunctionality. Nevertheless, it is well known that these materials imply severe environmental and human health impacts since these polymers are mostly petroleum-derived and non-degradable [12].

Alternatively, due to recent technological advances that have potentiated the effective extraction and further processability of natural raw materials into artificial fibres, man-made organic fibres have risen and progressively attracted attention as a greener alternative to synthetic ones. Cellulose, in particular, is the most abundant organic material on earth, and through chemical modifications can be employed for manufacturing cellulose derivatives and regenerated fibres [10]. Besides cellulosic fibres, new chemical routes have been opened to incorporate diverse and multifunctional natural-based raw materials into fibre composition. Moreover, new bio-based monomers as previously described in this article have been recently used to synthesise greener processable polymers. These materials could be derived from polysaccharides, proteins, lignin and other bioactive derivatives, and lipids extracted from renewable resources, as discussed in the following sub-sections.

### 4.1. Production Methods

This sub-section focuses on the methods usually employed to generate fibrous materials with the goal of valorising previously described biopolymers derived from biomass or biowaste and subsequent monomers.

Essentially, the methodology for producing fibrous materials should be compatible and adaptable with the polymeric composition and its inherent qualities, such as chemical structure, solubility, crystallinity, conductivity, and thermal and mechanical characteristics. It is well known that natural polymers most studied are derived from polysaccharides and proteins, which typically present lower thermal and mechanical properties than synthetic polymers. To address this constraint, biopolymeric compositions are often treated using wet-/dry-spinning and electrospinning. Melt-spinning is only performed on biopolymeric blends that have been functionalised with specified additives or contain graft-functionalised polymers.

Nowadays, diverse fibres or fibrous materials can be spun by drawing a melt or solution of a polymer, polymeric blend, or nanocomposite from a spinneret into a medium (quenching or water, coagulation bath) for solidification. This solidification medium may be air or a specific bath (water, quenching, or coagulation). In addition, the drawing step can be conducted by rollers and winders by high-velocity air stream or by electrostatic force [241]. Attending this recent diversity, this section will focus on a description of the most prevalent fibre fabrication methods, namely melt-spinning, electrospinning, dry-spinning, and wet/dry-jet-spinning.

Melt-spinning, also known as the extrusion method, is the most used method for manufacturing commercial synthetic fibres with multifunctional properties at a large scale. This is due to the simplicity of the production line, high spinning velocities, low production cost, solvent-free process, and environmental friendliness [241,242]. A melt-spinning setup includes a screw extruder, where a thermoplastic polymer or polymeric blend (in the form of pellets or chips) is homogenously melted and pressurised, a melt pump that controls the throughput rate, and a spin pack that ensures polymer filtering and distribution for the spinneret, in order to occur the filament formation. The extruded filaments are cooled (either into a quenching chamber or a water bath) and a spin finish is applied. Afterwards, the filaments move to the filament draw-down unit where they are drawn by several godets and collected on a bobbin through the winder [241]. Since this process involves thermal processing, a basic requirement is that the polymer or polymeric blend becomes fusible below its degradation temperature. Hence, not all monomers/polymers can be processed through this technique. Furthermore, certain additives are frequently combined with polymeric compositions, especially for biopolymers, to improve their thermal processability and provide better mechanical properties and durability of the obtained fibres [241]. Moreover, as presented in Table 3, several parameters related to polymer composition and processing conditions can critically affect the spinnability and quality of melt-spun fibres. For instance, bio-based polymers with small molecular weights and amorphous chemical structures can greatly influence the processing temperature. On the other hand, the difference between the extrusion speed and collecting speed could increase the degree of molecular orientation and crystallinity. Therefore, it may contribute to the improvement of mechanical performance [243].

Electrospinning is also a low-cost and effective approach to producing high-quality continuous fibres at the micrometre to the nanometre scale. This technique can be applied to a wide range of polymers (synthetic and natural), resulting in high interfibrous pore size and high specific surface area being able to be further functionalised [23,245,246,247,248,249,250,251,252,253]. The principle of this technology is based on the action of an electrical field that is applied to a polymeric solution. Following this, an accelerated liquid jet of polymeric solution is formed and, due to the solvents’ fast evaporation, the polymer’s solidification occurs and is then adequately collected. The spinnability and fibre quality can be affected by several parameters as follows: solution properties, such as concentration, molecular weight, conductivity, and viscosity; processing conditions, such as solvent, feeding rate, spinning distance, voltage, and temperature; and system design, for example, coaxial spinning and rotating collector [245,254,255].

Dry-spinning is a simple solution-spinning method that allows for the production of fibres with distinct compositions. This technology is based on the evaporation of the volatile solvents commonly used to obtain the polymer solution, also known as spinning dope [256]. So, in this method, the dope solution is injected/extruded through a spinneret setup, passing into hot air for quick solvent evaporation before the fibres are collected and submitted to drawing processing [243]. Besides this simplicity, some parameters could affect the structure and performance of dry-spun fibres (see Table 3). In this regard, it should be mentioned that these fibres could present low production efficiency, mostly due to their instant solidification, which may lead to voids and residual solvents within the fibre structure, compromising their mechanical performance [243,256].

On behalf of spinning processes using solidification solution, wet/dry-jet-spinning has risen as a promising low-cost fibre fabrication at the macroscale [257,258,259], since it is a variation in the widespread wet-spinning suitable for highly viscous polymeric solutions with thermal sensitivity. In wet-/dry-jet-spinning, the polymeric solution is injected/extruded into an air gap, which promotes the molecular orientation and an appropriate stretching of the polymeric jet before passing into a coagulation bath. In addition, the coagulated fibres are collected at variable speeds to promote their stretching. As in previous methods, different parameters can affect the quality of wet-/dry-spun fibres, but they generally exhibit better mechanical properties than melt-spun fibres due to their higher draw ratios [243].

### 4.2. Bio-Based Man-Made Fibres

Fibrous materials produced from natural polymers or biopolymers have received much interest in recent years due to their lower environmental impact in comparison with the dominant petroleum-derived polymers. Subsequently, MMOFs or bio-based man-made have a promising future in different fields since their molecular structure could be fine-tuned for textile, sportswear, healthcare, and protection applications using specific solvents and suitable fibre preparation and processing techniques [228,251].

**Figure 6:**
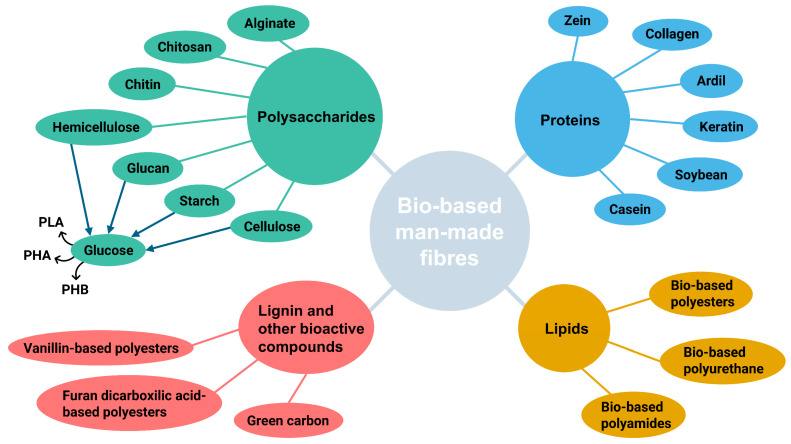
New routes to produce bio-based man-made fibres through biopolymers directly extracted from polysaccharide and protein fractions or via bio-based monomers involved in different polymer synthesis, which could be extracted from polysaccharides, lignin and other bioactive compounds, and lipids.

Hence, the subsequent sub-sections will focus on the main developments in creating polymeric formulations based on promising biocompounds for the manufacturing of fibres employing the previously mentioned spinning processes. To create high-quality, multifunctional man-made fibres suitable for a wide range of applications, methods for tuning the main biopolymer classes—polysaccharides, proteins, lignin, and lipid derivatives (Figure 6)—will be covered.

#### 4.2.1. Polysaccharides

**Figure 7:**
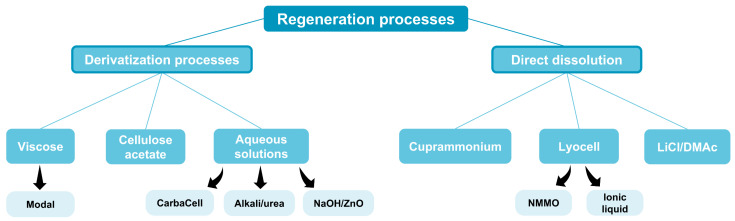
Schematic illustration with the most relevant processes used to obtain regenerated cellulosic fibres.

As previously described, different polysaccharides could be obtained from biomass/waste. Among them, cellulose is the most abundant and versatile polymer and is extensively exploited to develop fibres and diverse fibrous materials. In the context of the textile industry, cellulosic fibres derived from cotton or wood pulp prepared by wet-spinning are still playing an important role. Accordingly, different cellulosic solvent systems have been explored to develop regenerated cellulosic fibres (RCF), as it was extensively described by Shen et al. [11] and Sayyed et al. [12]. As illustrated in Figure 7, there are two approaches for obtaining regenerated fibres: through derivatisation processes that modify cellulose before its dissolution or by direct dissolution of cellulose.

From derivatisation processes, the viscose (NaOH/CS_2_) [12,258,260,261,262,263,264,265] and its derivative Modal^®^ [12], obtained from wood pulp, and cellulose acetate [266], obtained from both wood pulp and cotton litters, stand out as the most significant commercially applied methods for textiles and other applications. In this context, recent adjustments have been introduced to make these procedures more sustainable. TENCEL™ Lyocell fibres are viscose fibres produced in a resource-saving closed-loop process that allows for significant solvent recovery. On the other hand, TENCEL™ Modal’s production method is described as resource-efficient, with high rates of recovery and recycling than generic modal. Nevertheless, these two conventional methods still have detrimental environmental effects because they use the volatile and highly toxic carbon disulfide (CS2) in their solvent system; thus, new aqueous solution systems have emerged recently as greener, harmless, and rapid dissolution solvents. Some examples of these aqueous solutions are CarbaCell (known as Carbamate), a sodium hydroxide solution to dissolve cellulose carbamate [216,231,267,268,269,270,271,272,273,274], Alkali/urea [275,276,277,278], and sodium zincate solution (Biocelsol^®^) NaOH/ZnO [271,279].

On behalf of direct dissolution processes, cuprammonium (known as cupro or cuprammonium rayon) [12,280,281,282,283] consists of an expensive industrial process to obtain silky man-made fibres. Furthermore, lithium chloride/N,N-dimethylacetamide (LiCl/DMAc) [284,285] represent an organic solvent system recurrently used to dissolve cellulose and its derivatives. Addressing the environmental concerns involved in these direct dissolution processes, Lyocell processes (also known as Tencel^®^) [229,286,287,288,289] have recently emerged as advantageous environmentally friendly methods that could use N-Methylmorpholine-*N*-Oxide (NMMO) or ionic liquids [227,258,264,290].

Moreover, concerning the mechanical performance of these different regenerated cellulosic fibres compiled in Table 4, it should be mentioned that NMMO-based Lyocell fibres exhibited good fibre strength and other adequate physical properties, and are by far one of the most environment-friendly and non-hazardous cellulose regeneration process. In this sense, Lyocell fibres present clear advantages over conventional viscose or cupro fibres [12,287].

Aligned with new trends in manufacturing regenerated cellulosic fibres, the brand Orange Fiber was a pioneer in producing fabrics from citrus juice waste, owning a patented innovative process to obtain sustainable cellulosic fibres from orange pulp. In fact, Orange Fiber is a viscose fibre made of 100% natural cellulose obtained from citrus fruits waste, such as oranges, lemons, tangerines and grapefruits, through extraction processes that uses only ecological chemicals. This viscose fibre is biodegradable, and it is commonly called vegetable silk due to its silky and airy texture [291]. In addition, the recent partnership between Orange Fiber and the Lenzing group resulted in the first-ever TENCEL™ branded Lyocell fibre made of orange and wood pulp, which claims to provide high-quality comfort and performance, and enhanced sustainability in the textile industry [291].

Besides cellulose and its derivatives, other polysaccharides that play an important role in the structural support of diverse organisms, such as chitosan/chitin [96,99,110,251,257,292,293,294], starch [295,296], and alginate (SeaCell^®^ fibres) [297,298,299,300], have been increasingly incorporated into the composition of man-made fibres. As shown in Table 5, these biopolymers are recurrently used to produce fibres with advanced (bio)functionalities for textile and biomedical applications through wet-spinning or electrospinning.

Moreover, concerning the synthesis of biopolymers based on polysaccharide-derived monomers, it should be mentioned that polyhydroxyalkanoates (PHAs), unlike other polyesters, can be produced through bacterial fermentation of glucose. These polyesters are environmentally friendly, biodegradable, and biocompatible, and could represent an alternative to petroleum-based plastics, especially in the medical field. However, the main limitation associated with this process is the high production cost (e.g., 5–10 times higher than conventional plastic), which is mainly influenced by the yields of carbon sources, the fermentation process, and productivity [301,302].

Among a range of biodegradable polymers, polylactic acid (PLA) is an aliphatic polyester that presents excellent performance and physicochemical attributes; so, it has become the most used bioplastic in diverse fields, such as biomedical, textile, food packaging, and automotive [303]. This biopolymer is derived from lactic acid, which can be obtained from the fermentation of food crops rich in starch and cellulose (corn, wheat, or rice). After obtaining the lactic acid, PLA synthesis typically involves ring-opening polymerisation. This process necessitates the completion of numerous separation and purification stages, resulting in a high cost of PLA production [303,304].

Focusing on reducing the cost of those bioplastics, different waste materials have been re-purposed as carbon sources, namely whey from the dairy processing industry; wheat, rice bran, starch, sugarcane molasses, and vegetable oils from the food industry; and wastewater from different industries (textile, paper, and abattoir) [301,302].

#### 4.2.2. Proteins

The development of protein-based fibres, RPFs, also known as azlon fibres, occurred during the world wars (throughout the 20th century) when textile manufacturers looked at alternatives for natural fibres and considered food waste a valuable resource. In this sense, scientific and industrial advances resulted in the invention of Ardil, a RPF developed by the British company Imperial Chemical Industries from storage protein extracted from peanut waste (mainly arachin) through a patented process by wet-spinning. However, the discontinuation of regenerated protein fibres was mostly caused by the rise in high-performance petrochemical fibres [305].

Despite the failure of RDF Ardil, the research and development of RDFs remain active, and, in the market, some commercial examples are already available.

QMILK fibres are made using a unique upcycling method that uses a biopolymer derived from non-food milk proteins and 100% renewable raw materials (such as organic oils). It exhibits soft and smooth properties as silk while displaying natural antibacterial and flame-retardant effects and high hydrophilicity. In addition, these MMOFs claim to be completely biodegradable and compatible with natural fibres through thermo-bonding processes [306].

Using supramolecular technology, UMORFIL^®^ developed an upcycle yarn process that incorporates collagen peptide amino acid—which is extracted from aquaculture fish scale—into viscose, nylon, or polyester fibres to provide a range of bionic functional fibres that offer improved moisture retention, biodegradability and a soft touch [307].

Babysoy company features an innovative collection of sustainable and comfortable clothing for babies made with the soybean protein fibre. This azlon textile fibre is obtained by wet-spinning through a patented process where proteins are extracted from the leftover pulp of tofu or soy milk production. Essentially, this azlon fibre has gained attention because of its silky smooth and cashmere feel, superior moisture transmission for dryness, dimensional stability, natural flame resistance, and better UV protection when compared to cotton or bamboo fibre [308].

In the literature, some works have reported distinct strategies for the functionalisation of man-made fibres with plant-derived and animal-derived proteins. These compounds could provide structural support and enhance physiochemical properties and diverse surface functionalities. In Table 5, some relevant works containing plant-derived proteins are presented as follows: zein and other prolamins (hordein, gliadin) [248,253,300,309,310]; soybean protein [311]; animal-derived proteins, such as keratin [299,312,313,314,315], collagen [316,317,318], and gelatine [298,319], which are largely conducted with versatile electrospinning and few with standard wet-spinning methodology.

#### 4.2.3. Lignin and Other Bioactive Compounds

Lignin and derived phenolic compounds have attracted attention for bio-based polyester synthesis. Among other phenolic compounds, vanillin is a common molecule extracted on an industrial scale from lignin. It could be explored for the formulation of vanillin-based monomers (e.g., diols, diacids/diesters, hydroxyacids/hydroxyesters), as well as for the synthesis of vanillin-based polyesters [320].

Terpenoids are a diverse class of low-molecular-weight organic compounds synthesised as part of plant secondary metabolism. These compounds can be foreseen as a sustainable resource for obtaining terpene-derived monomers used to synthesise bio-based aliphatic polyesters [321,322,323].

Furan derivatives are also valuable compounds that can be extracted from different lignocellulosic biomass, providing key functional groups to synthesise diverse materials [324]. Particularly, bis(hydromethyl) furan or furan dicarboxylic acid (FDCA) has been produced at a large (as the example of Avantium [325]) and further explored for the synthesis of new bio-based polyesters. This could occur through reactions with other dicarboxylic acids or diols, such as furan dicarboxylic acid-based polyesters, which exhibit excellent thermal and mechanical properties [326,327,328].

Besides this biorefinery interest, some works in the literature describe the development of fibres with the incorporation of lignin as a valuable nanofiller. It usually provides structural support, flame retardancy, antibacterial, and anti-inflammatory properties, which is also a greener and cheaper strategy to produce high-performance carbon fibres (see Table 5).

#### 4.2.4. Lipids

In fact, the lipidic fraction of different lignocellulosic wastes and biomasses has been extensively studied as a valuable raw resource to provide bio-based monomers for the synthesis of new bio-based polymers, including polyesters, polyethylene, and polyamides. Similarly to petroleum-derived polymers, these new bio-based polymers might be processed using melt-spinning or electrospinning to produce fibres with a variety of (bio)functionalities for textile and biomedical applications. Regarding bio-based polyesters, recent developments in the preparation of bio-based monomers have provided the fast growth of new bio-based polyesters with tunable properties and a large range of application areas. Zhang et al. [320] complied with the recent advances in the field of bio-based polyesters (including aliphatic and aromatic) and the main aliphatic monomers derived from plant oils. The main compounds highlighted were ethylene glycol, 1,2-propanediol, 1,3-propanediol, 1,6-hexanediol, 1,10-hexanediol, azelaic acid, sebacic acid, dodecanedioic acid and glycerol [320].

Moreover, a new generation of polyurethane derived from renewable raw materials has risen to replace petroleum-based ones. Among the most common bio-based polyols applied for bio-polyurethane synthesis, vegetable oils (including castor oil, oleic acid, and linoleic acid) have been increasingly exploited due to their hydroxyl group composition [329,330]. For instance, as shown in Table 5, these bio-polyurethanes can be combined with other functional components to electrospun fibres with advanced functionalities for diverse applications. In the polyamide domain, due to their excellent intrinsic properties and industrial significance in a wide range of applications, recent efforts have been made to develop bio-based polyamides, also known as bionylons. PA6 and PA6.6 are the two most widely used polyamides, as they are responsible for the massive consumption of petroleum-based materials in their synthesis (around 10 million tonnes each year) [331,332]. Since the monomers commonly used in polyamide synthesis (e.g., dicarboxylic acids, diamines, and caprolactam) are highly dependent on petroleum chemical resources, it is urgent to find greener and bio-based monomers to reduce this environmental impact. For instance, some vegetable oils have been extensively studied for this application. Among them, castor oil has gained particular attention due to its high amount of ricinoleic acid, which provides a favourable source of bio-based dicarboxylic acids, such as undecylenic acid and sebacic acid [333].

Some examples of bio-based polyamides already on the market are as follows: PA4.10 and PA6.10, derived from sebacic acid; PA10.12 and PA10T, derived from decanediamine; and two fully bio-derived PA11 and PA10.10, which can be synthesised from ricinoleic acid and the melt polycondensation of sebacic and decanediamine [333,334,335,336,337]. In particular, the Rilsan^®^ PA11 supplied by Arkema is derived from ricinoleic acid obtained from castor oil (a bio-based content of around 98%) and presents excellent chemical resistance, easy processing, high dimensional stability and long-term durability suitable for different high-performance applications [338]. Furthermore, Radilon^®^ provides a wide range of polyamides with excellent mechanical and chemical resistance, including long-chain polyamides (Radilon^®^ D) produced from bio-derived PA6.10 made with 64% renewable source material [339]. Recently, Fulgar^®^ developed innovative high-performance and extra comfort bio-based yarns, EVO^®^, made by bio-polyamide polymerised with monomers partially or totally derived from castor oil [340].

Driven by the sustainability transition, the use of novel high-performance textile fibres has risen. The Bio-based Dyneema^®^ fibre, for example, uses a renewable bio-based feedstock (a by-product from wood pulp industry) supplied by sustainable forest management to produce green ethylene, making it the first bio-based high molecular polyethylene (HMPE) fibre. This more sustainable Dyneema^®^ fibre reduces the dependence on fossil fuel-based resources without compromising protection, comfort, or durability [341].

**Table 5:** Diverse man-made fibres produced at different scales, using natural polymers functionalised or combined with biofillers for distinct applications.

Composition	Spinning Method	Properties	Application	References
Chitosan + Graphene Chitosan + PVA + Graphene Chitosan + Cotton + Polyester + Tencel Chitin + Cellulose	Wet-spinning Electrospinning Wet-spinning Wet-, dry-spinning	Softness, biodegradability, thermal conductivity, antibacterial properties	Antibacterial and technical fibres	[292]
				[293]
				[294]
				[257]
Bacterial cellulose + Silica	Wet-spinning	Thermal insulation	Technical fibres	[342]
Starch + PVA + Glycerol	Wet-spinning	Biodegradability, skin friendly	Functional fibres	[295]
Starch + PLA Starch + PLGA Starch + Nanocellulose	Electrospinning	Hydrophobicity, biodegradability	Functional nanofibres	[296]
Keratin + Cellulose	Dry-jet wet-spinning	Flexibility and mechanical resistance	Precursor for carbon fibres	[312]
Keratin + Cellulose nanocrystals	Wet-spinning	Hierarchically structured fibres with shape-memory features	Functional fibres	[313]
Viscose + Zein	Wet-spinning	High mechanical performance, biodegradability	Technical fibres	[309]
Polyurethane + Keratin + AgNPs	Electrospinning	Biocompatibility and antibacterial properties	Nanofibrous mats for wound dressing	[314]
Poly(hydroxybutylate-co-hydroxyvalerate) + Keratin	Electrospinning	Biocompatibility, bioadhesiveness, biodegradability	Nanofibrous mats for wound dressing	[315]
Collagen + Nanohydroxyapatite	Electrospinning	Biocompatibility, bioadhesiveness, biodegradability	Nanofibrous mats for bone regeneration	[316]
PCL + Collagen	Electrospinning	Biocompatibility, bioadhesiveness, vascularisation	Biofunctionalised nanofibrous mats for tissue regeneration	[317]
PLGA + Collagen	Electrospinning	Biocompatibility, bioadhesiveness	Nanofibrous structures for tissue regeneration	[318]
Gelatin + Tyrosine	Electrospinning	Biocompatibility, bioadhesiveness	Nanofibrous mats for cartilage tissue regeneration	[319]
Zein	Electrospinning	Core–shell structure	Drug-loaded nanofibrous mats	[248]
Soybean protein	Wet-spinning	Controlled drug load and release delivery	Biofunctional fibres for drug delivery	[311]
Zein/Gliadin/Hordein	Electrospinning	Good mechanical properties, biocompatibility	Ultrafine fibres for biomedical applications	[253]
Alginate + Pectin + Gelatin + Glycerol	Wet-spinning	Monofilament is bioabsorbable and capable of drug delivery	Suture for biomedical applications	[298]
Keratin + Alginate	Wet-spinning	Dual crosslinked fibres suitable for complex braid forms	Flexible fibres	[299]
Zein + Alginate + Betanin + TiO_2_NPs	Electrospinning	Good mechanical performance, hydrophobicity, antibacterial properties	Nanofibres for food packaging	[300]
Regenerated cellulose + Cellulose diacetate/Cellulose acetate propionate/Cellulose acetate butyrate	Wet-spinning	Transparency, thermal and chemical stability	Biopolymeric optical fibres	[343]
Bio-based polyamide 56	Electrospinning	Good mechanical performance, antibacterial properties	Bionylon nanofibres for functional textiles	[336]
Bio-based polyamide 56	Melt-spinning	Good mechanical and thermal performance, flame-retardancy, biodegradability	Bionylon fibres for functional textiles	[337]
Bio-polyurethane + Triclosan + Cyclodextrin	Electrospinning	Good mechanical properties and antibacterial	Antibacterial nanofibrous materials	[329]
Zein + Hordein + Lignin	Electrospinning	Good electrochemical properties, hierarchical porous texture	Supercapacitors precursors for carbon fibres with flame-retardancy	[310]
Cellulose	Electrospinning	Reinforced mechanical and thermal properties	Biodegradable nanofibrous composites	[344]
Cellulose/cellulose acetate Cellulose acetate/polyurethane	Electrospinning	Light transparency and improved mechanical properties	Light transparent nanofibrous composites	[345,346,347,348]

## 5. Conclusions and Future Perspectives

In recent years, driven by growing concerns over sustainability and environmental challenges, there has been a significant surge in research and development focused on renewable-based polymers.

In this regard, among the abundant biomass/waste sources herein reported, the agroforestry and industrial lignocellulosic residues could be the preferred resources to obtain valuable building blocks—polysaccharides, proteins, lignin, and derived phenolic compounds and lipids—suitable for a wide range of biorefineries.

Attending to the complex hierarchical structure of diverse lignocellulosic resources, the efficient extraction of their major components (e.g., cellulose, hemicellulose, lignin, vegetable oil) could be challenging. Essentially, the processing of lignocellulosic materials typically relies on conventional extraction protocols that combine physical pre-treatments with alkali or acidic chemical methods, which exhibit enormous environmental impact. Consequently, these conventional chemical extractions started to be replaced by greener solvent systems that could also be effective in terms of extraction yields, such as organosolv, deep-eutectic, or ionic liquids extractions. Alternatively, biological methods could be conducted, providing a more selective bio-pulping. However, these last emergent ecofriendly extractions are expensive and excessively time-consuming, requiring further research and optimisation to be further implemented at a large scale.

Respecting the production of man-made fibres, chemical and technological developments observed in the commonly used production methods (e.g., melt-spinning, electrospinning, and wet-/dry-jet spinning) have contributed to a progressive improvement in the fibre quality obtained. Moreover, bio-based polymers such as PLA, PHA, PHB, bio-polyamides, biopolyesters, and bio-polyurethanes are being incorporated into man-made fibres. These can be combined with structural polysaccharides like cellulose, chitosan, starch, and alginate, as well as proteins such as keratin, collagen, and gelatin. This approach creates multiscale fibres with multifunctional properties, which could be applied to textiles, sportswear, biomedical uses, and protective gear. In this way, a new generation of man-made fibres, known as MMOFs, is emerging, which might be built up of a variety of bio-based polymers.

As it was demonstrated by the present review, biomass/waste sources have a high potential for replacing petroleum-based monomers. In this way, based on the biopolymers examined in this research, novel synthesis strategies have been developed that can reach all or almost all current polymers. However, these strategies still present certain limitations, such as the development of new synthesis pathways and the refinement of formulations to achieve the same attributes in biopolymers as we do in petroleum derivatives. Furthermore, if the performance of petroleum goods cannot be met, evaluate product adaptability and expectations for them. On the other hand, it may be intriguing to investigate strategies to lower the cost associated with the production of bio-derived polymers. Otherwise, it can be assumed that the high cost is a reasonable price to pay for sustainable bio-based polymers.

## Figures and Tables

**Table 1:** Lignocellulosic composition (wt%) of different biomass resources and their principal applications. Notation: abs and n.d. mean absence and not determined, respectively.

Biomass Resource	Cellulose (%)	Hemicellulose (%)	Pectin (%)	Lignin (%)	Applications	References
Hemp (stalks)	67–76	12–18	3–18	3–6	Textile, paper, food, cosmetics, packaging, biodiesel	[19]
Jute (stalks)	61–73	14–23	n.d.	12–16	Textile, packaging, biocomposites	[31]
Nettle (leaves, stem, roots)	54–88	4–10	1–4.1	5–9	Textile, food, biocomposites, biomedical	[79]
Juncus (stem)	40–64	20–28	n.d.	6–19	Biocomposites	[51]
Giant reed (leaves, stem, rhizome)	21–38	11–42	n.d.	13–32	Paper, biorefineries, bioplastics, biocomposites	[52]
Johnsongrass (leaves, stem)	40	36	n.d.	10	Bioenergy, biorefineries, biocomposites	[59]
*Opuntia ficus-indica* L. (stem)	53	11	n.d.	4.8	Paper, food, pharmaceutical biocomposites	[80]
*Agave americana* L. (leaves)	59.5	17.4	6.80	13.6	Biocomposites	[67]
*Aloe vera* (leaves)	64.9	25.1	n.d.	4.1	Textile, cosmetic, pharmaceutical, biocomposites	[69]
*Chlorella vulgaris* (microalgae)	10–47.5	n.d.	n.d.	n.d.	Biorefinery, bioplastics, biocomposites	[78]
*Chlorella pyrenoidosa* (microalgae)	15.4	31	n.d.	n.d.	Biorefinery, bioplastics, biocomposites	[81]
*Cladophora rupestris* (macroalgae)	28.5	abs	abs	n.d.	Biorefinery, bioplastics, biocomposites	[82]
*Chaetomorpha melagonium* (macroalgae)	41	n.d.	n.d.	n.d.	Biorefinery, bioplastics, biocomposites	[82]
*Laminaria digitata* (macroalgae)	20	n.d	n.d.	n.d.	Biorefinery, bioplastics, biocomposites	[82]
*Halidrys siliquosa* (macroalgae)	14	n.d.	n.d	n.d.	Biorefinery, bioplastics, biocomposites	[82]
*Ulva* sp.	40.7	7.1	n.d.	7.9	Paper, biorefinery	[83]
Water hyacinth	12.8–14.9	24–27.5	n.d.	5.9–14.3	Textile, paper, biocomposites	[84,85]
Waterweeds	18.8	5	n.d.	2.3	Biosynthesis of nanoparticles	[86,87,88]

**Table 2:** Lignocellulosic composition of different biowaste resources and their principal applications.

Biowaste Product		Cellulose (%)	Hemicellulose (%)	Pectin (%)	Lignin (%)	Applications	References
Hardwood	Poplar	50–53	26–29	-	15.5–16	Paper, biorefineries, biocomposites	[126,127]
	Eucalyptus	54	18	-	21.5		[126]
Softwood	Pine	45–50	25–35	-	25–35	Biorefineries, biocomposites	[126,127]
	Spruce	46	23	-	28		[126]
Cereals	Wheat straw	35–39	23–30	-	12–16	Biorefineries, PHA	[120,126,127]
	Corn stalk	35–40	17–35	-	7–18		
	Barley straw	36–43	24–33	-	6.3–10		
Fruits	Apple pomace	40–44	19–24	9–12	15–20	Biorefineries, bio-fertiliser, biofiller, PHA, xanthan gum	[128]
	Orange pulp	25.3	5.3	15.7–16.3	2.2–3.0		[128]
	Peach pomace	29–30	19–20	21–24	5–6		[128]
	Coconut coir	44	22	-	33		[129]
	Banana waste	13	15	-	14		[130]
	Spent coffee ground	12	39	-	24		[131,132,133]
Vegetables	Soybean straw	25	12	-	18	Biorefineries, bio-fertiliser, biofiller, PHA, PLA, xanthan gum	[120]
	Tomato pomace	9–19	5–12	7.5	3–36		[128,134]
	Potato pulp	17–22	14	2.2	2.6		[128,134]
	Olive pomace	19–37	22–27	16–17	26–40		[134,135]
	Carrot pomace	28–52	7–12	2–4	18–32		[134,136]
Marine animals	Crustacean shells	-	-	-	-	Chitin and chitosan extraction	[137,138,139,140,141]
Abattoir	Meat and poultry waste	-	-	-	-	Collagen, gelatine, keratin, PHA	[142,143,144,145,146,147,148,149,150,151,152,153,154,155]
Dairy	Milk and cheese waste	-	-	-	-	PHA, xanthan gum	[156,157,158,159,160,161,162,163,164]
Eucalyptus black liquor	Paper industry	-	1–2	-	40–42	Biorefineries, biogas, PHA, carboxylic acids	[165,166,167,168]
Discarded textile	Pre-consumer and post-consumer	-	-	-	-	Extraction of cellulose derivatives, polyester monomers, glucose and amino acids, biorefineries, fillers	[169,170,171,172,173,174,175,176,177,178,179,180]

**Table 3:** Principal fibre fabrication methods and their major solution and processing parameters (adapted from [241,243,244]).

Fabrication Method	Composition	Process
Melt-spinning	Polymer molecular structure Polydispersity Crystallinity Melting temperature Glass transition Decomposition temperature Enhancing and functional additives	Screw extruder (type and velocity) Spin pack design (spinneret) Heating temperature Extrusion speed Draw ratio Coagulation bath (composition and temperature) Take-up speed Environmental temperature and humidity
Electrospinning	Polymer concentration Viscosity Conductivity Solvent evaporation rate Molecular weight	Flow rate Applied voltage Tip to collector distance Collector types Environmental temperature and humidity
Dry-spinning	Polymer concentration Molecular weight Solvent	Spinneret size Drawing ratio Take-up speed Environmental temperature and humidity
Wet/Dry-jet-spinning	Polymer concentration Molecular weight Solvent Viscosity	Spinneret size Air gap Coagulation bath Drawing ratio Take-up speed Environmental temperature and humidity

**Table 4:** Mechanical properties exhibited by common regenerated cellulosic fibres.

Process	Spinning Method	Tenacity (cN/dTex)	Elongation (%)	References
Viscose (NaOH/CS_2_)	Wet-spinning	1.80 (dry)	18.00 (dry)	[262]
		1.00 (wet)	21.60 (wet)	
		2.07 (dry)	19.20 (dry)	[263]
		0.75 (wet)	12.50 (wet)	
		2.15	22.60	[264]
		1.77–2.30 (dry)	17.00–25.00 (dry)	[258]
		0.88–1.32 (wet)	21.00–30.00 (wet)	[265]
Cellulose acetate	Air-jet spinning	1.06–1.24 (dry)	25.00–35.00 (dry)	[266]
		0.57–0.66 (wet)	35.00–45.00 (wet)	
Alkali/urea	Wet-spinning	3.50 (dry)	8.00 (dry)	[275]
		2.50 (wet)	8.70 (wet)	
		3.43	10.20	[276]
		2.20	1.90	[277]
		1.90	2.00	[278]
NaOH/ZnO	Wet-spinning	2.36 (dry)	15.90 (dry)	[271]
		0.73 (wet)	17.80 (wet)	
		2.58	12.10	[279]
Cuprammonium	Wet-spinning	2.00 (dry)	10.00 (dry)	[280]
		1.00 (wet)	20.00 (wet)	
		1.50–2.39 (dry)	12.00–13.00 (dry)	
		1.41–1.50 (wet)	26.00–27.00 (wet)	
LiCl/DMAc	Wet-spinning	4.23	7.76	[284]
		1.68–3.27 (dry)	6.00–12.00 (dry)	[285]
		0.53–2.30 (wet)	8.00–18.00 (wet)	
Ionic liquids	Dry-jet wet-spinning	3.60 (dry)	10.20 (dry)	[290]
		2.70 (wet)	12.40 (wet)	
		3.85	7.90	[258]
		3.09–3.62 (dry)	7.00–8.00 (dry)	[227]
		2.03–2.74 (wet)	9.00–12.00 (wet)	[264]
NMMO	Dry-jet wet-spinning	4.90	4.00	[259]
		4.30	13.10	[264]
		3.97–4.42 (dry)	11.00–16.00 (dry)	[286]
		3.44–3.53 (wet)	16.00–18.00 (wet)	[258]

## References

[1] Gunaalan K., Fabbri E., Capolupo M. (2020). The Hidden Threat of Plastic Leachates: A Critical Review on Their Impacts on Aquatic Organisms. Water Res..

[2] Andrady A.L., Neal M.A. (2009). Applications and Societal Benefits of Plastics. Philos. Trans. R. Soc. B Biol. Sci..

[3] PlasticsEurope Plastics–The Fast Facts 2023. https://plasticseurope.org/knowledge-hub/plastics-the-fast-facts-2023/.

[4] Franzellitti S., Canesi L., Auguste M., Wathsala R.H.G.R., Fabbri E. (2019). Microplastic Exposure and Effects in Aquatic Organisms: A Physiological Perspective. Environ. Toxicol. Pharmacol..

[5] Gallo F., Fossi C., Weber R., Santillo D., Sousa J., Ingram I., Nadal A., Romano D. (2018). Marine Litter Plastics and Microplastics and Their Toxic Chemicals Components: The Need for Urgent Preventive Measures. Environ. Sci. Eur..

[6] Xue B., Zhang L., Li R., Wang Y., Guo J., Yu K., Wang S. (2020). Underestimated Microplastic Pollution Derived from Fishery Activities and “Hidden” in Deep Sediment. Environ. Sci. Technol..

[7] Wang X., Zhu Q., Yan X., Wang Y., Liao C., Jiang G. (2020). A Review of Organophosphate Flame Retardants and Plasticizers in the Environment: Analysis, Occurrence and Risk Assessment. Sci. Total Environ..

[8] Schiavo S., Oliviero M., Chiavarini S., Manzo S. (2020). Adverse Effects of Oxo-Degradable Plastic Leachates in Freshwater Environment. Environ. Sci. Pollut. Res..

[9] Hahladakis J.N., Velis C.A., Weber R., Iacovidou E., Purnell P. (2018). An Overview of Chemical Additives Present in Plastics: Migration, Release, Fate and Environmental Impact during Their Use, Disposal and Recycling. J. Hazard. Mater..

[10] Quintana E., Valls C., Roncero M.B. (2024). Dissolving-Grade Pulp: A Sustainable Source for Fiber Production. Wood Sci. Technol..

[11] Shen H., Sun T., Zhou J. (2023). Recent Progress in Regenerated Cellulose Fibers by Wet Spinning. Macromol. Mater. Eng..

[12] Sayyed A.J., Deshmukh N.A., Pinjari D.V. (2019). A Critical Review of Manufacturing Processes Used in Regenerated Cellulosic Fibres: Viscose, Cellulose Acetate, Cuprammonium, LiCl/DMAc, Ionic Liquids, and NMMO Based Lyocell. Cellulose.

[13] Aswathi Mohan A., Robert Antony A., Greeshma K., Yun J.-H., Ramanan R., Kim H.-S. (2022). Algal Biopolymers as Sustainable Resources for a Net-Zero Carbon Bioeconomy. Bioresour. Technol..

[14] Moura P., Henriques J., Alexandre J., Oliveira A.C., Abreu M., Gírio F., Catarino J. (2022). Sustainable Value Methodology to Compare the Performance of Conversion Technologies for the Production of Electricity and Heat, Energy Vectors and Biofuels from Waste Biomass. Clean. Waste Syst..

[15] European Union Directive 2009/28/EC of the European Parliament and of the Council of 23 April 2009 on the Promotion of the Use of Energy from Renewable Sources and Amending and Subsequently Repealing Directives 2001/77/EC and 2003/30/EC. https://eur-lex.europa.eu/legal-content/EN/TXT/PDF/?uri=CELEX:32009L0028&from=EN.

[16] U.S. Energy Information Administration Biomass Explained. https://www.eia.gov/energyexplained/biomass/.

[17] Gold S., Seuring S. (2011). Supply Chain and Logistics Issues of Bio-Energy Production. J. Clean. Prod..

[18] Zhang Z., Huang J., Yao Y., Peters G., Macdonald B., La Rosa A.D., Wang Z., Scherer L. (2023). Environmental Impacts of Cotton and Opportunities for Improvement. Nat. Rev. Earth Environ..

[19] Promhuad K., Srisa A., San H., Laorenza Y., Wongphan P., Sodsai J., Tansin K., Phromphen P., Chartvivatpornchai N., Ngoenchai P. (2022). Applications of Hemp Polymers and Extracts in Food, Textile and Packaging: A Review. Polymers.

[20] Tutek K., Masek A. (2022). Hemp and Its Derivatives as a Universal Industrial Raw Material (with Particular Emphasis on the Polymer Industry)—A Review. Materials.

[21] Singha S., Mahmutovic M., Zamalloa C., Stragier L., Verstraete W., Svagan A.J., Das O., Hedenqvist M.S. (2021). Novel Bioplastic from Single Cell Protein as a Potential Packaging Material. ACS Sustain. Chem. Eng..

[22] Moscariello C., Matassa S., Esposito G., Papirio S. (2021). From Residue to Resource: The Multifaceted Environmental and Bioeconomy Potential of Industrial Hemp (*Cannabis sativa* L.). Resour. Conserv. Recycl..

[23] Mouro C., Gomes A.P., Gouveia I.C. (2023). From Hemp Waste to Bioactive Nanofiber Composites: Deep Eutectic Solvents and Electrospinning in Upcycling Endeavors. Gels.

[24] Ahmed B., Wu Q., Lin H., Gwon J., Negulescu I., Cameron B. (2022). Degumming of Hemp Fibers Using Combined Microwave Energy and Deep Eutectic Solvent Treatment. Ind. Crops Prod..

[25] Palanikumar K., Natarajan E., Markandan K., Ang C.K., Franz G. (2023). Targeted Pre-Treatment of Hemp Fibers and the Effect on Mechanical Properties of Polymer Composites. Fibers.

[26] Ma J., Ma Q., Fu J., Shen G., Meng C. (2023). Deep Eutectic Solvent Degumming of Hemp Fiber: Key Factors Influencing Fiber Property and Its Mechanism. Ind. Crops Prod..

[27] Viscusi G., Lamberti E., Rosaria Acocella M., Gorrasi G. (2023). Production of Electrospun Hybrid Membranes Based on Polyamide 6 Reinforced with Hemp Fibers Dissolved in 1-Ethyl-3-Methylimidazolium Dicyanamide Ionic Liquid. J. Mol. Liq..

[28] Pacaphol K., Aht-Ong D. (2017). Preparation of Hemp Nanofibers from Agricultural Waste by Mechanical Defibrillation in Water. J. Clean. Prod..

[29] Dhali K., Daver F., Cass P., Adhikari B. (2021). Isolation and Characterization of Cellulose Nanomaterials from Jute Bast Fibers. J. Environ. Chem. Eng..

[30] Biswas A., Dey S., Huang S., Deng Y., Birhanie Z.M., Zhang J., Akhter D., Liu L., Li D. (2022). A Comprehensive Review of *C. capsularis* and *C. olitorius*: A Source of Nutrition, Essential Phytoconstituents and Pharmacological Activities. Antioxidants.

[31] Shahinur S., Sayeed M.M.A., Hasan M., Sayem A.S.M., Haider J., Ura S. (2022). Current Development and Future Perspective on Natural Jute Fibers and Their Biocomposites. Polymers.

[32] Chakraborty A., Sarkar D., Satya P., Karmakar P.G., Singh N.K. (2015). Pathways Associated with Lignin Biosynthesis in Lignomaniac Jute Fibres. Mol. Genet. Genom..

[33] Eichhorn S.J., Davies G.R. (2006). Modelling the Crystalline Deformation of Native and Regenerated Cellulose. Cellulose.

[34] Guan Y., Li F., Wang Y., Guo M., Hou J. (2024). “Reservoir-Law” Synergistic Reinforcement of Electrostatic Spun Polylactic Acid Composites with Cellulose Nanocrystals and 2-Hydroxypropyl-β-Cyclodextrin for Intelligent Bioactive Food Packaging. Int. J. Biol. Macromol..

[35] Das K., Ray D., Banerjee C., Bandyopadhyay N.R., Sahoo S., Mohanty A.K., Misra M. (2010). Physicomechanical and Thermal Properties of Jute-Nanofiber-Reinforced Biocopolyester Composites. Ind. Eng. Chem. Res..

[36] Maiti S., Ray D., Mitra D., Misra M. (2012). Study of Compostable Behavior of Jute Nano Fiber Reinforced Biocopolyester Composites in Aerobic Compost Environment. J. Appl. Polym. Sci..

[37] Khan G.M.A., Haque M.A., Terano M., Alam M.S. (2014). Graft Polycondensation of Microfibrillated Jute Cellulose with Oligo (L-lactic acid) and Its Properties. J. Appl. Polym. Sci..

[38] Meshram J.H., Palit P. (2013). On the Role of Cell Wall Lignin in Determining the Fineness of Jute Fibre. Acta Physiol. Plant.

[39] Del Río J.C., Rencoret J., Marques G., Li J., Gellerstedt G., Jiménez-Barbero J., Martínez A.T., Gutiérrez A. (2009). Structural Characterization of the Lignin from Jute (*Corchorus capsularis*) Fibers. J. Agric. Food Chem..

[40] Liu J., Li X., Li M., Zheng Y. (2022). Lignin Biorefinery: Lignin Source, Isolation, Characterization, and Bioconversion. Advances in Bioenergy.

[41] Sfiligoj Smole M., Hribernik S., Kurečič M., Urbanek Krajnc A., Kreže T., Stana Kleinschek K. (2019). Preparation of Cellulose Nanocrystals CNC from Nettle, Weeping Willow, Balm-Leaved Archangel, Lucerne and Spanish Broom. Surface Properties of Non-Conventional Cellulose Fibres.

[42] Phanthong P., Reubroycharoen P., Hao X., Xu G., Abudula A., Guan G. (2018). Nanocellulose: Extraction and Application. Carbon Resour. Convers..

[43] Kregiel D., Pawlikowska E., Antolak H. (2018). *Urtica* spp.: Ordinary Plants with Extraordinary Properties. Molecules.

[44] Grauso L., de Falco B., Lanzotti V., Motti R. (2020). Stinging Nettle, *Urtica dioica* L.: Botanical, Phytochemical and Pharmacological Overview. Phytochem. Rev..

[45] Upton R. (2013). Stinging Nettles Leaf (*Urtica dioica* L.): Extraordinary Vegetable Medicine. J. Herb. Med..

[46] Bogard F., Bach T., Abbes B., Bliard C., Maalouf C., Bogard V., Beaumont F., Polidori G. (2022). A Comparative Review of Nettle and Ramie Fiber and Their Use in Biocomposites, Particularly with a PLA Matrix. J. Nat. Fibers.

[47] Kassab Z., Syafri E., Tamraoui Y., Hannache H., Qaiss A.E.K., El Achaby M. (2020). Characteristics of Sulfated and Carboxylated Cellulose Nanocrystals Extracted from Juncus Plant Stems. Int. J. Biol. Macromol..

[48] Kassab Z., Mansouri S., Tamraoui Y., Sehaqui H., Hannache H., Qaiss A.E.K., El Achaby M. (2020). Identifying Juncus Plant as Viable Source for the Production of Micro- and Nano-Cellulose Fibers: Application for PVA Composite Materials Development. Ind. Crops Prod..

[49] Kassab Z., Daoudi H., Salim M.H., El Idrissi El Hassani C., Abdellaoui Y., El Achaby M. (2024). Process-Structure-Property Relationships of Cellulose Nanocrystals Derived from Juncus Effusus Stems on ҡ-Carrageenan-Based Bio-Nanocomposite Films. Int. J. Biol. Macromol..

[50] Benali M., Oulmekki A., Toyir J. (2023). The Impact of the Alkali-Bleaching Treatment on the Isolation of Natural Cellulosic Fibers from *Juncus effesus* L. Plant. Fibers Polym..

[51] Naili H., Jelidi A., Limam O., Khiari R. (2017). Extraction Process Optimization of Juncus Plant Fibers for Its Use in a Green Composite. Ind. Crops Prod..

[52] Ortega Z., Bolaji I., Suárez L., Cunningham E. (2023). A Review of the Use of Giant Reed (*Arundo donax* L.) in the Biorefineries Context. Rev. Chem. Eng..

[53] Zhang D., Jiang Q., Liang D., Huang S., Liao J. (2021). The Potential Application of Giant Reed (*Arundo donax*) in Ecological Remediation. Front. Environ. Sci..

[54] Di Fidio N., Fulignati S., De Bari I., Antonetti C., Raspolli Galletti A.M. (2020). Optimisation of Glucose and Levulinic Acid Production from the Cellulose Fraction of Giant Reed (*Arundo donax* L.) Performed in the Presence of Ferric Chloride under Microwave Heating. Bioresour. Technol..

[55] Eroğlu Ö., Çetin N.S., Narlıoğlu N., Yan W. (2023). Plastic/Fiber Composite Using Recycled Polypropylene and Fibers from *Sorghum halepense* L.. Bioresources.

[56] Iqbal A., Badshah S.L., Alves J.L.F., da Silva J.C.G., Di Domenico M. (2022). An Insight into the Thermokinetics of the Pyrolysis of Invasive Grass *Sorghum Halepense* towards Its Bioenergy Potential. Biomass Convers. Biorefin..

[57] Peter A., Žlabur J.Š., Šurić J., Voća S., Purgar D.D., Pezo L., Voća N. (2021). Invasive Plant Species Biomass—Evaluation of Functional Value. Molecules.

[58] Scordia D., Testa G., Cosentino S.L. (2014). Perennial Grasses as Lignocellulosic Feedstock for Second-Generation Bioethanol Production in Mediterranean Environment. Ital. J. Agron..

[59] Scordia D., Testa G., Copani V., Patanè C., Cosentino S.L. (2017). Lignocellulosic Biomass Production of Mediterranean Wild Accessions (*Oryzopsis Miliacea*, *Cymbopogon Hirtus*, *Sorghum Halepense* and *Saccharum Spontaneum*) in a Semi-Arid Environment. Field Crops Res..

[60] Zamboi A., Fraterrigo Garofalo S., Tommasi T., Fino D. (2024). Optimization of Ultrasounds Assisted Extraction of Polysaccharides from Cladodes of Opuntia Ficus-Indica Using Response Surface Methodology. Sustain. Chem. Pharm..

[61] Albergamo A., Potortí A.G., Di Bella G., Amor N.B., Lo Vecchio G., Nava V., Rando R., Ben Mansour H., Lo Turco V. (2022). Chemical Characterization of Different Products from the Tunisian Opuntia *Ficus-Indica* (L.) Mill. Foods.

[62] De Andrade Vieira É., Tribuzy de Magalhães Cordeiro A.M. (2023). Bioprospecting and Potential of Cactus Mucilages: A Bibliometric Review. Food Chem..

[63] Sevgi A., Özçelik M., Yılmaz T. (2022). Extraction, Characterization, and Rheology of *Opuntia ficus indica* Cladode Polysaccharides. J. Food Process Preserv..

[64] Bezazi A., Belaadi A., Bourchak M., Scarpa F., Boba K. (2014). Novel Extraction Techniques, Chemical and Mechanical Characterisation of *Agave americana* L. Natural Fibres. Compos. B Eng..

[65] Sathiamurthi P., Karthi Vinith K.S., Sathishkumar T.P., Arunkumar S., Anaamalaai A.S. (2021). Fiber Extraction and Mechanical Properties of Agave Americana/Kenaf Fiber Reinforced Hybrid Epoxy Composite. Mater. Today Proc..

[66] Evdokimova O.L., Alves C.S., Krsmanović Whiffen R.M., Ortega Z., Tomás H., Rodrigues J. (2021). Cytocompatible Cellulose Nanofibers from Invasive Plant Species *Agave americana* L. and *Ricinus communis* L.: A Renewable Green Source of Highly Crystalline Nanocellulose. J. Zhejiang Univ. Sci. B.

[67] Gebretsadik T.T., Tesfay A.H., Gebru A.G., Assayehegn E., Desta Y.H., Gebremedhin K.H., Gebrehiwet H., Teklemedhin T.B. (2023). Characterization and Comparative Insights on *Agave americana* and *Agave sisalana* Leaf Fibers for High-Performance Applications. J. Nat. Fibers.

[68] Saha J., Mondal M.I.H., Ahmed F., Rahman M. (2023). Extraction, Characterization and Functionality Assessment of Aloe Vera, Chitosan and Silk Sericin. Arab. J. Chem..

[69] Dehouche N., Idres C., Kaci M., Zembouai I., Bruzaud S. (2020). Effects of Various Surface Treatments on Aloe Vera Fibers Used as Reinforcement in Poly(3-Hydroxybutyrate-Co-3-Hydroxyhexanoate) (PHBHHx) Biocomposites. Polym. Degrad. Stab..

[70] Balaji A.N., Nagarajan K.J. (2017). Characterization of Alkali Treated and Untreated New Cellulosic Fiber from Saharan Aloe Vera Cactus Leaves. Carbohydr. Polym..

[71] Tennakoon P., Chandika P., Yi M., Jung W.-K. (2023). Marine-Derived Biopolymers as Potential Bioplastics, an Eco-Friendly Alternative. iScience.

[72] Bose I., Nousheen, Roy S., Yaduvanshi P., Sharma S., Chandel V., Biswas D. (2023). Unveiling the Potential of Marine Biopolymers: Sources, Classification, and Diverse Food Applications. Materials.

[73] Zanchetta E., Damergi E., Patel B., Borgmeyer T., Pick H., Pulgarin A., Ludwig C. (2021). Algal Cellulose, Production and Potential Use in Plastics: Challenges and Opportunities. Algal Res..

[74] Ross I.L., Shah S., Hankamer B., Amiralian N. (2021). Microalgal Nanocellulose–Opportunities for a Circular Bioeconomy. Trends Plant Sci..

[75] Klassen V., Blifernez-Klassen O., Bax J., Kruse O. (2020). Wastewater-Borne Microalga *Chlamydomonas* sp.: A Robust Chassis for Efficient Biomass and Biomethane Production Applying Low-N Cultivation Strategy. Bioresour. Technol..

[76] Zhu S., Feng S., Xu Z., Qin L., Shang C., Feng P., Wang Z., Yuan Z. (2019). Cultivation of *Chlorella Vulgaris* on Unsterilized Dairy-Derived Liquid Digestate for Simultaneous Biofuels Feedstock Production and Pollutant Removal. Bioresour. Technol..

[77] Kothari R., Pathak V.V., Kumar V., Singh D.P. (2012). Experimental Study for Growth Potential of Unicellular Alga *Chlorella Pyrenoidosa* on Dairy Waste Water: An Integrated Approach for Treatment and Biofuel Production. Bioresour. Technol..

[78] Aguirre A., Bassi A. (2013). Investigation of Biomass Concentration, Lipid Production, and Cellulose Content in *Chlorella Vulgaris* Cultures Using Response Surface Methodology. Biotechnol. Bioeng..

[79] Viotti C., Albrecht K., Amaducci S., Bardos P., Bertheau C., Blaudez D., Bothe L., Cazaux D., Ferrarini A., Govilas J. (2022). Nettle, a Long-Known Fiber Plant with New Perspectives. Materials.

[80] Mannai F., Ammar M., Yanez J.G., Elaloui E., Moussaoui Y. (2018). Alkaline Delignification of Cactus Fibres for Pulp and Papermaking Applications. J. Polym. Environ..

[81] Northcote D.H., Goulding K.J., Horne R.W. (1960). The Chemical Composition and Structure of the Cell Wall of *Hydrodictyon africanum* Yaman. Biochem. J..

[82] Cronshaw J., Myers A., Preston R.D. (1958). A Chemical and Physical Investigation of the Cell Walls of Some Marine Algae. Biochim. Biophys. Acta.

[83] Moral A., Aguado R., Castelló R., Tijero A., Ballesteros M. (2019). Potential Use of Green Alga *Ulva* sp. for Papermaking. Bioresources.

[84] Zhou W., Zhu D., Langdon A., Li L., Liao S., Tan L. (2009). The Structure Characterization of Cellulose Xanthogenate Derived from the Straw of *Eichhornia crassipes*. Bioresour. Technol..

[85] Sari N.H., Suteja, Rangappa S.M., Siengchin S. (2023). A Review on Cellulose Fibers from *Eichornia crassipes*: Synthesis, Modification, Properties and Their Composites. J. Nat. Fibers.

[86] Gallegos D., Wedwitschka H., Moeller L., Weinrich S., Zehnsdorf A., Nelles M., Stinner W. (2018). Mixed Silage of Elodea and Wheat Straw as a Substrate for Energy Production in Anaerobic Digestion Plants. Energy Sustain. Soc..

[87] Poorter H., Bergkotte M. (1992). Chemical Composition of 24 Wild Species Differing in Relative Growth Rate. Plant Cell Environ..

[88] Anbu P., Gopinath S.C.B., Salimi M.N., Letchumanan I., Subramaniam S. (2022). Green Synthesized Strontium Oxide Nanoparticles by *Elodea Canadensis* Extract and Their Antibacterial Activity. J. Nanostruct. Chem..

[89] Thiripura Sundari M., Ramesh A. (2012). Isolation and Characterization of Cellulose Nanofibers from the Aquatic Weed Water Hyacinth—*Eichhornia crassipes*. Carbohydr. Polym..

[90] Ajithram A., Winowlin Jappes J.T., Brintha N.C. (2021). Water Hyacinth (*Eichhornia crassipes*) Natural Composite Extraction Methods and Properties—A Review. Mater. Today Proc..

[91] Din S., Hamid S., Yaseen A., Yatoo A.M., Ali S., Shamim K., Mahdi W.A., Alshehri S., Rehman M.U., Shah W.A. (2022). Isolation and Characterization of Flavonoid Naringenin and Evaluation of Cytotoxic and Biological Efficacy of Water Lilly (*Nymphaea mexicana* Zucc.). Plants.

[92] Hsu C.-L., Fang S.-C., Yen G.-C. (2013). Anti-Inflammatory Effects of Phenolic Compounds Isolated from the Flowers of *Nymphaea mexicana* Zucc. Food Funct..

[93] Bai C., Su F., Zhang W., Kuang H. (2023). A Systematic Review on the Research Progress on Polysaccharides from Fungal Traditional Chinese Medicine. Molecules.

[94] Wang B., Shi Y., Lu H., Chen Q. (2023). A Critical Review of Fungal Proteins: Emerging Preparation Technology, Active Efficacy and Food Application. Trends Food Sci. Technol..

[95] Wang W., Tan J., Nima L., Sang Y., Cai X., Xue H. (2022). Polysaccharides from Fungi: A Review on Their Extraction, Purification, Structural Features, and Biological Activities. Food Chem. X.

[96] Islam S., Bhuiyan M.A.R., Islam M.N. (2017). Chitin and Chitosan: Structure, Properties and Applications in Biomedical Engineering. J. Polym. Environ..

[97] Hisham F., Maziati Akmal M.H., Ahmad F., Ahmad K., Samat N. (2024). Biopolymer Chitosan: Potential Sources, Extraction Methods, and Emerging Applications. Ain Shams Eng. J..

[98] Muñoz G., Valencia C., Valderruten N., Ruiz-Durántez E., Zuluaga F. (2015). Extraction of Chitosan from *Aspergillus niger* mycelium and Synthesis of Hydrogels for Controlled Release of Betahistine. React. Funct. Polym..

[99] Perrin N., Mohammadkhani G., Homayouni Moghadam F., Delattre C., Zamani A. (2022). Biocompatible Fibers from Fungal and Shrimp Chitosans for Suture Application. Curr. Res. Biotechnol..

[100] Svensson S.E., Ferreira J.A., Hakkarainen M., Adolfsson K.H., Zamani A. (2021). Fungal Textiles: Wet Spinning of Fungal Microfibers to Produce Monofilament Yarns. Sustain. Mater. Technol..

[101] Xu C., Wang F., Guan S., Wang L. (2024). β-Glucans Obtained from Fungus for Wound Healing: A Review. Carbohydr. Polym..

[102] Arslan N.P., Dawar P., Albayrak S., Doymus M., Azad F., Esim N., Taskin M. (2023). Fungi-Derived Natural Antioxidants. Crit. Rev. Food Sci. Nutr..

[103] Zhang X., Zhang T., Zhao Y., Jiang L., Sui X. (2024). Structural, Extraction and Safety Aspects of Novel Alternative Proteins from Different Sources. Food Chem..

[104] Spranghers T., Ottoboni M., Klootwijk C., Ovyn A., Deboosere S., De Meulenaer B., Michiels J., Eeckhout M., De Clercq P., De Smet S. (2017). Nutritional Composition of Black Soldier Fly (*Hermetia illucens*) Prepupae Reared on Different Organic Waste Substrates. J. Sci. Food Agric..

[105] Mohan K., Ganesan A.R., Muralisankar T., Jayakumar R., Sathishkumar P., Uthayakumar V., Chandirasekar R., Revathi N. (2020). Recent Insights into the Extraction, Characterization, and Bioactivities of Chitin and Chitosan from Insects. Trends Food Sci. Technol..

[106] Queiroz L.S., Nogueira Silva N.F., Jessen F., Mohammadifar M.A., Stephani R., Fernandes de Carvalho A., Perrone Í.T., Casanova F. (2023). Edible Insect as an Alternative Protein Source: A Review on the Chemistry and Functionalities of Proteins under Different Processing Methods. Heliyon.

[107] Van Huis A. (2016). Edible Insects Are the Future?. Proc. Nutr. Soc..

[108] Santiago L.A., Queiroz L.S., Tavares G.M., Feyissa A.H., Silva N.F., Casanova F. (2024). Edible Insect Proteins: How Can They Be a Driver for Food Innovation?. Curr. Opin. Food Sci..

[109] Saenz-Mendoza A.I., Zamudio-Flores P.B., García-Anaya M.C., Velasco C.R., Acosta-Muñiz C.H., Espino-Díaz M., Tirado-Gallegos J.M., Hernández-González M., Vela-Gutiérrez G., Salgado-Delgado R. (2023). Insects as a Potential Source of Chitin and Chitosan: Physicochemical, Morphological and Structural Characterization—A Review. Emir. J. Food Agric..

[110] Philibert T., Lee B.H., Fabien N. (2017). Current Status and New Perspectives on Chitin and Chitosan as Functional Biopolymers. Appl. Biochem. Biotechnol..

[111] Machado S.S.N., da Silva J.B.A., Nascimento R.Q., Lemos P.V.F., de Jesus Assis D., Marcelino H.R., de Souza Ferreira E., Cardoso L.G., Pereira J.D., Santana J.S. (2024). Insect Residues as an Alternative and Promising Source for the Extraction of Chitin and Chitosan. Int. J. Biol. Macromol..

[112] Hahn T., Tafi E., Paul A., Salvia R., Falabella P., Zibek S. (2020). Current State of Chitin Purification and Chitosan Production from Insects. J. Chem. Technol. Biotechnol..

[113] Brigode C., Hobbi P., Jafari H., Verwilghen F., Baeten E., Shavandi A. (2020). Isolation and Physicochemical Properties of Chitin Polymer from Insect Farm Side Stream as a New Source of Renewable Biopolymer. J. Clean. Prod..

[114] Rehman K.U., Hollah C., Wiesotzki K., Heinz V., Aganovic K., Rehman R.U., Petrusan J.-I., Zheng L., Zhang J., Sohail S. (2023). Insect-Derived Chitin and Chitosan: A Still Unexploited Resource for the Edible Insect Sector. Sustainability.

[115] Nunes L.J.R., Loureiro L.M.E.F., Sá L.C.R., Silva H.F.C. (2020). Waste Recovery through Thermochemical Conversion Technologies: A Case Study with Several Portuguese Agroforestry By-Products. Clean. Technol..

[116] Gaspar M.C., Mendes C.V.T., Pinela S.R., Moreira R., Carvalho M.G.V.S., Quina M.J., Braga M.E.M., Portugal A.T. (2019). Assessment of Agroforestry Residues: Their Potential within the Biorefinery Context. ACS Sustain. Chem. Eng..

[117] Gupta J., Kumari M., Mishra A., Swati, Akram M., Thakur I.S. (2022). Agro-Forestry Waste Management—A Review. Chemosphere.

[118] Shao H., Zhao H., Xie J., Qi J., Shupe T.F. (2019). Agricultural and Forest Residues towards Renewable Chemicals and Materials Using Microwave Liquefaction. Int. J. Polym. Sci..

[119] Bangar S.P., Whiteside W.S., Kajla P., Tavassoli M. (2023). Value Addition of Rice Straw Cellulose Fibers as a Reinforcer in Packaging Applications. Int. J. Biol. Macromol..

[120] Thorenz A., Wietschel L., Stindt D., Tuma A. (2018). Assessment of Agroforestry Residue Potentials for the Bioeconomy in the European Union. J. Clean. Prod..

[121] Kang K., Klinghoffer N.B., ElGhamrawy I., Berruti F. (2021). Thermochemical Conversion of Agroforestry Biomass and Solid Waste Using Decentralized and Mobile Systems for Renewable Energy and Products. Renew. Sustain. Energy Rev..

[122] Caetano N.S., Caldeira D., Martins A.A., Mata T.M. (2017). Valorisation of Spent Coffee Grounds: Production of Biodiesel via Enzymatic Catalysis with Ethanol and a Co-Solvent. Waste Biomass Valorization.

[123] Liu C., Luan P., Li Q., Cheng Z., Xiang P., Liu D., Hou Y., Yang Y., Zhu H. (2021). Biopolymers Derived from Trees as Sustainable Multifunctional Materials: A Review. Adv. Mater..

[124] Ferrari F., Striani R., Fico D., Alam M.M., Greco A., Esposito Corcione C. (2022). An Overview on Wood Waste Valorization as Biopolymers and Biocomposites: Definition, Classification, Production, Properties and Applications. Polymers.

[125] Sirviö J.A., Mikola M., Ahola J., Heiskanen J.P., Filonenko S., Ämmälä A. (2023). Highly Effective Fractionation Chemistry to Overcome the Recalcitrance of Softwood Lignocellulose. Carbohydr. Polym..

[126] Isikgor F.H., Becer C.R. (2015). Lignocellulosic Biomass: A Sustainable Platform for the Production of Bio-Based Chemicals and Polymers. Polym. Chem..

[127] Cai J., He Y., Yu X., Banks S.W., Yang Y., Zhang X., Yu Y., Liu R., Bridgwater A.V. (2017). Review of Physicochemical Properties and Analytical Characterization of Lignocellulosic Biomass. Renew. Sustain. Energy Rev..

[128] Plakantonaki S., Roussis I., Bilalis D., Priniotakis G. (2023). Dietary Fiber from Plant-Based Food Wastes: A Comprehensive Approach to Cereal, Fruit, and Vegetable Waste Valorization. Processes.

[129] Subhedar P.B., Ray P., Gogate P.R. (2018). Intensification of Delignification and Subsequent Hydrolysis for the Fermentable Sugar Production from Lignocellulosic Biomass Using Ultrasonic Irradiation. Ultrason. Sonochem..

[130] Kumar A.K., Sharma S. (2017). Recent Updates on Different Methods of Pretreatment of Lignocellulosic Feedstocks: A Review. Bioresour. Bioprocess..

[131] Tapangnoi P., Sae-Oui P., Naebpetch W., Siriwong C. (2022). Preparation of Purified Spent Coffee Ground and Its Reinforcement in Natural Rubber Composite. Arab. J. Chem..

[132] Ballesteros L.F., Teixeira J.A., Mussatto S.I. (2014). Chemical, Functional, and Structural Properties of Spent Coffee Grounds and Coffee Silverskin. Food Bioproc Technol..

[133] Singh T.A., Pal N., Sharma P., Passari A.K. (2023). Spent Coffee Ground: Transformation from Environmental Burden into Valuable Bioactive Metabolites. Rev. Environ. Sci. Biotechnol..

[134] Toushik S.H., Lee K., Lee J., Kim K. (2017). Functional Applications of Lignocellulolytic Enzymes in the Fruit and Vegetable Processing Industries. J. Food Sci..

[135] Álvarez A., Cachero S., González-Sánchez C., Montejo-Bernardo J., Pizarro C., Bueno J.L. (2018). Novel Method for Holocellulose Analysis of Non-Woody Biomass Wastes. Carbohydr. Polym..

[136] Nawirska A., Kwaśniewska M. (2005). Dietary Fibre Fractions from Fruit and Vegetable Processing Waste. Food Chem..

[137] Prameela K., Venkatesh K., Vani K.D., Sudesh Kumar E., Mohan C.M. (2017). Eco-Friendly Extraction of Biopolymer Chitin and Carotenoids from Shrimp Waste. IOP Conf. Ser. Mater. Sci. Eng..

[138] Kaya M., Baran T., Karaarslan M. (2015). A New Method for Fast Chitin Extraction from Shells of Crab, Crayfish and Shrimp. Nat. Prod. Res..

[139] Pakizeh M., Moradi A., Ghassemi T. (2021). Chemical Extraction and Modification of Chitin and Chitosan from Shrimp Shells. Eur. Polym. J..

[140] Premasudha P., Vanathi P., Abirami M. (2017). Extraction and Characterization of Chitosan from Crustacean Waste: A Constructive Waste Management Approach. Int. J. Sci. Res..

[141] Ifuku S., Nogi M., Abe K., Yoshioka M., Morimoto M., Saimoto H., Yano H. (2009). Preparation of Chitin Nanofibers with a Uniform Width as α-Chitin from Crab Shells. Biomacromolecules.

[142] Ideia P., Pinto J., Ferreira R., Figueiredo L., Spínola V., Castilho P.C. (2020). Fish Processing Industry Residues: A Review of Valuable Products Extraction and Characterization Methods. Waste Biomass Valorization.

[143] Senthilkumar N., Chowdhury S., Sanpui P. (2023). Extraction of Keratin from Keratinous Wastes: Current Status and Future Directions. J. Mater. Cycles Waste Manag..

[144] Arunmozhivarman K. (2017). Extraction and Molecular Characterization of Collagen from Poultry Meat Processing By-Product (Chicken Skin). Int. J. Pure Appl. Biosci..

[145] Matinong A.M.E., Chisti Y., Pickering K.L., Haverkamp R.G. (2022). Collagen Extraction from Animal Skin. Biology.

[146] Manimegalai N.P., Ramanathan G., Gunasekaran D., Jeyakumar G.F.S., Sivagnanam U.T. (2022). Cardinal Acuity on the Extraction and Characterization of Soluble Collagen from the Underutilized Abattoir Junks for Clinical Demands. Process Biochem..

[147] Sockalingam K., Abdullah H.Z. (2015). Extraction and Characterization of Gelatin Biopolymer from Black Tilapia (*Oreochromis mossambicus*) Scales. AIP Conf. Proc..

[148] Rather J.A., Akhter N., Ashraf Q.S., Mir S.A., Makroo H.A., Majid D., Barba F.J., Khaneghah A.M., Dar B.N. (2022). A Comprehensive Review on Gelatin: Understanding Impact of the Sources, Extraction Methods, and Modifications on Potential Packaging Applications. Food Packag. Shelf Life.

[149] Alipal J., Mohd Pu’ad N.A.S., Lee T.C., Nayan N.H.M., Sahari N., Basri H., Idris M.I., Abdullah H.Z. (2021). A Review of Gelatin: Properties, Sources, Process, Applications, and Commercialisation. Mater. Today Proc..

[150] Vineis C., Varesano A., Varchi G., Aluigi A. (2019). Extraction and Characterization of Keratin from Different Biomasses. Keratin as a Protein Biopolymer.

[151] Chukwunonso Ossai I., Shahul Hamid F., Hassan A. (2022). Valorisation of Keratinous Wastes: A Sustainable Approach towards a Circular Economy. Waste Manag..

[152] Anbesaw M.S. (2022). Bioconversion of Keratin Wastes Using Keratinolytic Microorganisms to Generate Value-Added Products. Int. J. Biomater..

[153] Yukesh Kannah R., Dinesh Kumar M., Kavitha S., Rajesh Banu J., Kumar Tyagi V., Rajaguru P., Kumar G. (2022). Production and Recovery of Polyhydroxyalkanoates (PHA) from Waste Streams—A Review. Bioresour. Technol..

[154] Titz M., Kettl K.-H., Shahzad K., Koller M., Schnitzer H., Narodoslawsky M. (2012). Process Optimization for Efficient Biomediated PHA Production from Animal-Based Waste Streams. Clean. Technol. Environ. Policy.

[155] Muhr A., Rechberger E.M., Salerno A., Reiterer A., Malli K., Strohmeier K., Schober S., Mittelbach M., Koller M. (2013). Novel Description of Mcl-PHA Biosynthesis by Pseudomonas Chlororaphis from Animal-Derived Waste. J. Biotechnol..

[156] Sar T., Harirchi S., Ramezani M., Bulkan G., Akbas M.Y., Pandey A., Taherzadeh M.J. (2022). Potential Utilization of Dairy Industries By-Products and Wastes through Microbial Processes: A Critical Review. Sci. Total Environ..

[157] Ahmad T., Aadil R.M., Ahmed H., Rahman U.U., Soares B.C.V., Souza S.L.Q., Pimentel T.C., Scudino H., Guimarães J.T., Esmerino E.A. (2019). Treatment and Utilization of Dairy Industrial Waste: A Review. Trends Food Sci. Technol..

[158] Wu Z., Yin H., Liu W., Huang D., Hu N., Yang C., Zhao X. (2020). Xanthan Gum Assisted Foam Fractionation for the Recovery of Casein from the Dairy Wastewater. Prep. Biochem. Biotechnol..

[159] Cancella M.J., Cerqueira A.F.L.W., da Costa Teodoro L., Pereira J.R., da Costa Ludwig Z.M., de Carvalho Anjos V., Denadai Â.M.L., Húngaro H.M., Rodarte M.P. (2024). Xanthan Gum Produced from Milk Permeate and Deproteinized Cheese Whey: A Comparative Analysis with Commercial Xanthan Gums. Biocatal. Agric. Biotechnol..

[160] Costa C., Azoia N.G., Coelho L., Freixo R., Batista P., Pintado M. (2021). Proteins Derived from the Dairy Losses and By-Products as Raw Materials for Non-Food Applications. Foods.

[161] Galante R., Cunha F., Fangueiro R. (2023). Extraction and Properties of Casein Biopolymer from Milk. Handbook of Natural Polymers, Volume 1.

[162] Palaniraj A., Jayaraman V. (2011). Production, Recovery and Applications of Xanthan Gum by Xanthomonas Campestris. J. Food Eng..

[163] Colombo B., Pepè Sciarria T., Reis M., Scaglia B., Adani F. (2016). Polyhydroxyalkanoates (PHAs) Production from Fermented Cheese Whey by Using a Mixed Microbial Culture. Bioresour. Technol..

[164] Pais J., Serafim L.S., Freitas F., Reis M.A.M. (2016). Conversion of Cheese Whey into Poly(3-Hydroxybutyrate-Co-3-Hydroxyvalerate) by Haloferax Mediterranei. New Biotechnol..

[165] Bajpai P. (2018). Biermann’s Handbook of Pulp and Paper.

[166] Reyes L., Nikitine C., Vilcocq L., Fongarland P. (2020). Green Is the New Black—A Review of Technologies for Carboxylic Acid Recovery from Black Liquor. Green Chem..

[167] Viel M., Collet F., Lanos C. (2020). Effect of Compaction on Multi-Physical Properties of Hemp-Black Liquor Composites. J. Mater. Res. Technol..

[168] Morya R., Kumar M., Tyagi I., Kumar Pandey A., Park J., Raj T., Sirohi R., Kumar V., Kim S.-H. (2022). Recent Advances in Black Liquor Valorization. Bioresour. Technol..

[169] Stanescu M.D. (2021). State of the Art of Post-Consumer Textile Waste Upcycling to Reach the Zero Waste Milestone. Environ. Sci. Pollut. Res..

[170] Wang S., Salmon S. (2022). Progress toward Circularity of Polyester and Cotton Textiles. Sustain. Chem..

[171] Dissanayake D.G.K., Weerasinghe D.U. (2021). Fabric Waste Recycling: A Systematic Review of Methods, Applications, and Challenges. Mater. Circ. Econ..

[172] Pensupa N., Leu S.-Y., Hu Y., Du C., Liu H., Jing H., Wang H., Lin C.S.K. (2017). Recent Trends in Sustainable Textile Waste Recycling Methods: Current Situation and Future Prospects. Top. Curr. Chem..

[173] Costa C., Viana A., Silva C., Marques E.F., Azoia N.G. (2022). Recycling of Textile Wastes, by Acid Hydrolysis, into New Cellulosic Raw Materials. Waste Manag..

[174] Wang H., Kaur G., Pensupa N., Uisan K., Du C., Yang X., Lin C.S.K. (2018). Textile Waste Valorization Using Submerged Filamentous Fungal Fermentation. Process Saf. Environ. Prot..

[175] Navone L., Moffitt K., Hansen K.-A., Blinco J., Payne A., Speight R. (2020). Closing the Textile Loop: Enzymatic Fibre Separation and Recycling of Wool/Polyester Fabric Blends. Waste Manag..

[176] Li X., Hu Y., Du C., Lin C.S.K. (2019). Recovery of Glucose and Polyester from Textile Waste by Enzymatic Hydrolysis. Waste Biomass Valorization.

[177] Kaabel S., Arciszewski J., Borchers T.H., Therien J.P.D., Friščić T., Auclair K. (2023). Solid-State Enzymatic Hydrolysis of Mixed PET/Cotton Textiles. ChemSusChem.

[178] Zebec Ž., Poberžnik M., Lobnik A. (2022). Enzymatic Hydrolysis of Textile and Cardboard Waste as a Glucose Source for the Production of Limonene in *Escherichia coli*. Life.

[179] Li X., Zhang M., Luo J., Zhang S., Yang X., Igalavithana A.D., Ok Y.S., Tsang D.C.W., Lin C.S.K. (2019). Efficient Succinic Acid Production Using a Biochar-Treated Textile Waste Hydrolysate in an in Situ Fibrous Bed Bioreactor. Biochem. Eng. J..

[180] Haslinger S., Hummel M., Anghelescu-Hakala A., Määttänen M., Sixta H. (2019). Upcycling of Cotton Polyester Blended Textile Waste to New Man-Made Cellulose Fibers. Waste Manag..

[181] Rao J., Gao H., Guan Y., Li W., Liu Q. (2019). Fabrication of Hemicelluloses Films with Enhanced Mechanical Properties by Graphene Oxide for Humidity Sensing. Carbohydr. Polym..

[182] Kun D., Pukánszky B. (2017). Polymer/Lignin Blends: Interactions, Properties, Applications. Eur. Polym. J..

[183] Schlee P., Hosseinaei O., O’ Keefe C.A., Mostazo-López M.J., Cazorla-Amorós D., Herou S., Tomani P., Grey C.P., Titirici M.-M. (2020). Hardwood versus Softwood Kraft Lignin–Precursor-Product Relationships in the Manufacture of Porous Carbon Nanofibers for Supercapacitors. J. Mater. Chem. A Mater..

[184] Worku L.A., Bachheti A., Bachheti R.K., Rodrigues Reis C.E., Chandel A.K. (2023). Agricultural Residues as Raw Materials for Pulp and Paper Production: Overview and Applications on Membrane Fabrication. Membranes.

[185] Ranganathan S., Dutta S., Moses J.A., Anandharamakrishnan C. (2020). Utilization of Food Waste Streams for the Production of Biopolymers. Heliyon.

[186] Arun K.B., Madhavan A., Sindhu R., Binod P., Pandey A., Reshmy R., Sirohi R. (2020). Remodeling Agro-Industrial and Food Wastes into Value-Added Bioactives and Biopolymers. Ind. Crops Prod..

[187] Shewry P.R., Tatham A.S. (1990). The Prolamin Storage Proteins of Cereal Seeds: Structure and Evolution. Biochem. J..

[188] Szymańska-Chargot M., Chylińska M., Gdula K., Kozioł A., Zdunek A. (2017). Isolation and Characterization of Cellulose from Different Fruit and Vegetable Pomaces. Polymers.

[189] Tapia-Blácido D.R., Maniglia B.C., Martelli-Tosi M. (2017). Biopolymers from Sugarcane and Soybean Lignocellulosic Biomass. Sustainable Polymers from Biomass.

[190] Obruca S., Petrik S., Benesova P., Svoboda Z., Eremka L., Marova I. (2014). Utilization of Oil Extracted from Spent Coffee Grounds for Sustainable Production of Polyhydroxyalkanoates. Appl. Microbiol. Biotechnol..

[191] Aslan A.K.H.N., Ali M.D.M., Morad N.A., Tamunaidu P. (2016). Polyhydroxyalkanoates Production from Waste Biomass. IOP Conf. Ser. Earth Environ. Sci..

[192] Karmee S.K. (2018). A Spent Coffee Grounds Based Biorefinery for the Production of Biofuels, Biopolymers, Antioxidants and Biocomposites. Waste Manag..

[193] Amiri H., Aghbashlo M., Sharma M., Gaffey J., Manning L., Moosavi Basri S.M., Kennedy J.F., Gupta V.K., Tabatabaei M. (2022). Chitin and Chitosan Derived from Crustacean Waste Valorization Streams Can Support Food Systems and the UN Sustainable Development Goals. Nat. Food.

[194] Food and Agriculture Organization of the United Nations (FAO) (2020). World Food and Agriculture—Statistical Yearbook 2020.

[195] Chokshi K., Pancha I., Ghosh A., Mishra S. (2016). Microalgal Biomass Generation by Phycoremediation of Dairy Industry Wastewater: An Integrated Approach towards Sustainable Biofuel Production. Bioresour. Technol..

[196] Porwal H.J., Mane A.V., Velhal S.G. (2015). Biodegradation of Dairy Effluent by Using Microbial Isolates Obtained from Activated Sludge. Water Resour. Ind..

[197] Braghiroli F.L., Passarini L. (2020). Valorization of Biomass Residues from Forest Operations and Wood Manufacturing Presents a Wide Range of Sustainable and Innovative Possibilities. Curr. For. Rep..

[198] Kasavan S., Yusoff S., Guan N.C., Zaman N.S.K., Fakri M.F.R. (2021). Global Trends of Textile Waste Research from 2005 to 2020 Using Bibliometric Analysis. Environ. Sci. Pollut. Res..

[199] Tang K.H.D. (2023). State of the Art in Textile Waste Management: A Review. Textiles.

[200] Tomovska E., Jordeva S., Trajković D., Zafirova K. (2016). Attitudes towards Managing Post-Industrial Apparel Cuttings Waste. J. Text. Inst..

[201] Shukla A., Kumar D., Girdhar M., Kumar A., Goyal A., Malik T., Mohan A. (2023). Strategies of Pretreatment of Feedstocks for Optimized Bioethanol Production: Distinct and Integrated Approaches. Biotechnol. Biofuels Bioprod..

[202] Padhi S., Singh A., Routray W. (2023). Nanocellulose from Agro-Waste: A Comprehensive Review of Extraction Methods and Applications. Rev. Environ. Sci. Biotechnol..

[203] Basbasan A.J., Hararak B., Winotapun C., Wanmolee W., Leelaphiwat P., Boonruang K., Chinsirikul W., Chonhenchob V. (2022). Emerging Challenges on Viability and Commercialization of Lignin in Biobased Polymers for Food Packaging: A Review. Food Packag. Shelf Life.

[204] Michelin M., Gomes D.G., Romaní A., Polizeli M.D.L.T., Teixeira J.A. (2020). Nanocellulose Production: Exploring the Enzymatic Route and Residues of Pulp and Paper Industry. Molecules.

[205] Bai L., Liu L., Esquivel M., Tardy B.L., Huan S., Niu X., Liu S., Yang G., Fan Y., Rojas O.J. (2022). Nanochitin: Chemistry, Structure, Assembly, and Applications. Chem. Rev..

[206] Giteru S.G., Ramsey D.H., Hou Y., Cong L., Mohan A., Bekhit A.E.A. (2023). Wool Keratin as a Novel Alternative Protein: A Comprehensive Review of Extraction, Purification, Nutrition, Safety, and Food Applications. Compr. Rev. Food Sci. Food Saf..

[207] Liu D., Smagghe G., Liu T.-X. (2023). Interactions between Entomopathogenic Fungi and Insects and Prospects with Glycans. J. Fungi.

[208] Guo X., Jiang Z., Li H., Li W. (2015). Production of Recycled Cellulose Fibers from Waste Paper via Ultrasonic Wave Processing. J. Appl. Polym. Sci..

[209] Calcio Gaudino E., Grillo G., Tabasso S., Stevanato L., Cravotto G., Marjamaa K., Pihlajaniemi V., Koivula A., Aro N., Uusitalo J. (2022). Optimization of Ultrasound Pretreatment and Enzymatic Hydrolysis of Wheat Straw: From Lab to Semi-Industrial Scale. J. Clean. Prod..

[210] González-Balderas R.M., Orta Ledesma M.T., Santana I., Felix M., Bengoechea C. (2023). *Desmodesmus* sp. from Biowaste to Produce Electrospinning Membranes: Effect of Ultrasounds and Ozone Pre-Treatments. J. Environ. Chem. Eng..

[211] Han W., Geng Y. (2023). Optimization and Characterization of Cellulose Extraction from Olive Pomace. Cellulose.

[212] Avelino F., da Silva K.T., de Souza Filho M.D.S.M., Mazzetto S.E., Lomonaco D. (2018). Microwave-Assisted Organosolv Extraction of Coconut Shell Lignin by Brønsted and Lewis Acids Catalysts. J. Clean. Prod..

[213] Marciano S.J., Avelino F., da Silva L.R.R., Mazzetto S.E., Lomonaco D. (2022). Microwave-Assisted Phosphorylation of Organosolv Lignin: New Bio-Additives for Improvement of Epoxy Resins Performance. Biomass Convers. Biorefin..

[214] Yan Y., Zhang C., Lin Q., Wang X., Cheng B., Li H., Ren J. (2018). Microwave-Assisted Oxalic Acid Pretreatment for the Enhancing of Enzyme Hydrolysis in the Production of Xylose and Arabinose from Bagasse. Molecules.

[215] Avelino F., Silva K.T., Mazzetto S.E., Lomonaco D. (2019). Tailor-Made Organosolv Lignins from Coconut Wastes: Effects of Green Solvents in Microwave-Assisted Processes upon Their Structure and Antioxidant Activities. Bioresour. Technol. Rep..

[216] Guo Y., Zhou J., Wang Y., Zhang L., Lin X. (2010). An Efficient Transformation of Cellulose into Cellulose Carbamates Assisted by Microwave Irradiation. Cellulose.

[217] Baksi S., Saha D., Saha S., Sarkar U., Basu D., Kuniyal J.C. (2023). Pre-Treatment of Lignocellulosic Biomass: Review of Various Physico-Chemical and Biological Methods Influencing the Extent of Biomass Depolymerization. Int. J. Environ. Sci. Technol..

[218] Prasetyo I., Permatasari P.R., Laksmana W.T., Rochmadi R., Oh W.-C., Ariyanto T. (2020). Lignin Refinery Using Organosolv Process for Nanoporous Carbon Synthesis. Molecules.

[219] Vieira F., Santana H.E.P., Silva D.P., Ruzene D.S. (2023). A Bibliometric Description of Organosolv Pretreatment for Coconut Waste Valorization. Bioenergy Res..

[220] Sidiras D., Politi D., Giakoumakis G., Salapa I. (2022). Simulation and Optimization of Organosolv Based Lignocellulosic Biomass Refinery: A Review. Bioresour. Technol..

[221] Ferreira J.A., Taherzadeh M.J. (2020). Improving the Economy of Lignocellulose-Based Biorefineries with Organosolv Pretreatment. Bioresour. Technol..

[222] Joy S.P., Krishnan C. (2022). Modified Organosolv Pretreatment for Improved Cellulosic Ethanol Production from Sorghum Biomass. Ind. Crops Prod..

[223] Ding R., Wu H., Thunga M., Bowler N., Kessler M.R. (2016). Processing and Characterization of Low-Cost Electrospun Carbon Fibers from Organosolv Lignin/Polyacrylonitrile Blends. Carbon.

[224] Chopda R., Ferreira J.A., Taherzadeh M.J. (2020). Biorefining Oat Husks into High-Quality Lignin and Enzymatically Digestible Cellulose with Acid-Catalyzed Ethanol Organosolv Pretreatment. Processes.

[225] Sar T., Arifa V.H., Hilmy M.R., Ferreira J.A., Wikandari R., Millati R., Taherzadeh M.J. (2022). Organosolv Pretreatment of Oat Husk Using Oxalic Acid as an Alternative Organic Acid and Its Potential Applications in Biorefinery. Biomass Convers. Biorefin..

[226] Kim T.H., Kwak H., Kim T.H., Oh K.K. (2020). Extraction Behaviors of Lignin and Hemicellulose-Derived Sugars During Organosolv Fractionation of Agricultural Residues Using a Bench-Scale Ball Milling Reactor. Energies.

[227] Kosan B., Michels C., Meister F. (2008). Dissolution and Forming of Cellulose with Ionic Liquids. Cellulose.

[228] Gough C.R., Rivera-Galletti A., Cowan D.A., Salas-de la Cruz D., Hu X. (2020). Protein and Polysaccharide-Based Fiber Materials Generated from Ionic Liquids: A Review. Molecules.

[229] Mohd N., Draman S.F.S., Salleh M.S.N., Yusof N.B. (2017). Dissolution of Cellulose in Ionic Liquid: A Review. AIP Conf. Proc..

[230] Taokaew S., Kriangkrai W. (2022). Recent Progress in Processing Cellulose Using Ionic Liquids as Solvents. Polysaccharides.

[231] Willberg-Keyriläinen P., Hiltunen J., Ropponen J. (2018). Production of Cellulose Carbamate Using Urea-Based Deep Eutectic Solvents. Cellulose.

[232] Abolore R.S., Jaiswal S., Jaiswal A.K. (2024). Green and Sustainable Pretreatment Methods for Cellulose Extraction from Lignocellulosic Biomass and Its Applications: A Review. Carbohydr. Polym. Technol. Appl..

[233] Rao J., Lv Z., Chen G., Peng F. (2023). Hemicellulose: Structure, Chemical Modification, and Application. Prog. Polym. Sci..

[234] Chen W.-H., Nižetić S., Sirohi R., Huang Z., Luque R., Papadopoulos A.M., Sakthivel R., Phuong Nguyen X., Tuan Hoang A. (2022). Liquid Hot Water as Sustainable Biomass Pretreatment Technique for Bioenergy Production: A Review. Bioresour. Technol..

[235] Wu Z., Peng K., Zhang Y., Wang M., Yong C., Chen L., Qu P., Huang H., Sun E., Pan M. (2022). Lignocellulose Dissociation with Biological Pretreatment towards the Biochemical Platform: A Review. Mater. Today Bio.

[236] Liu X., Jiang Y., Qin C., Yang S., Song X., Wang S., Li K. (2018). Enzyme-Assisted Mechanical Grinding for Cellulose Nanofibers from Bagasse: Energy Consumption and Nanofiber Characteristics. Cellulose.

[237] Ferdeș M., Dincă M.N., Moiceanu G., Zăbavă B.Ș., Paraschiv G. (2020). Microorganisms and Enzymes Used in the Biological Pretreatment of the Substrate to Enhance Biogas Production: A Review. Sustainability.

[238] Andlar M., Rezić T., Marđetko N., Kracher D., Ludwig R., Šantek B. (2018). Lignocellulose Degradation: An Overview of Fungi and Fungal Enzymes Involved in Lignocellulose Degradation. Eng. Life Sci..

[239] Pavithra S., Jayaprakash J., Gummadi S.N., Giri Dev V.R. (2023). Assessment of Process Integration Approach for Coir Biosoftening and Lignin-modifying Enzyme Production from Agro Residues. Biofuels Bioprod. Biorefin..

[240] Sánchez-Corzo L.D., Álvarez-Gutiérrez P.E., Meza-Gordillo R., Villalobos-Maldonado J.J., Enciso-Pinto S., Enciso-Sáenz S. (2021). Lignocellulolytic Enzyme Production from Wood Rot Fungi Collected in Chiapas, Mexico, and Their Growth on Lignocellulosic Material. J. Fungi.

[241] Hufenus R., Yan Y., Dauner M., Kikutani T. (2020). Melt-Spun Fibers for Textile Applications. Materials.

[242] Chen L., Pan D., He H. (2019). Morphology Development of Polymer Blend Fibers along Spinning Line. Fibers.

[243] Jin Y., Lin J., Cheng Y., Lu C. (2021). Lignin-Based High-Performance Fibers by Textile Spinning Techniques. Materials.

[244] DeFrates K.G., Moore R., Borgesi J., Lin G., Mulderig T., Beachley V., Hu X. (2018). Protein-Based Fiber Materials in Medicine: A Review. Nanomaterials.

[245] Teo W.-E., Inai R., Ramakrishna S. (2011). Technological Advances in Electrospinning of Nanofibers. Sci. Technol. Adv. Mater..

[246] Abdu M.T., Abuhasel K.A., Alquraish M., Nagy S., Khodir S., Ali A.A. (2023). Selected Natural Fibers and Their Electrospinning. J. Polym. Res..

[247] Salas C., Ago M., Lucia L.A., Rojas O.J. (2014). Synthesis of Soy Protein–Lignin Nanofibers by Solution Electrospinning. React. Funct. Polym..

[248] Huang W., Zou T., Li S., Jing J., Xia X., Liu X. (2013). Drug-Loaded Zein Nanofibers Prepared Using a Modified Coaxial Electrospinning Process. AAPS PharmSciTech.

[249] Ruiz-Rosas R., Bedia J., Lallave M., Loscertales I.G., Barrero A., Rodríguez-Mirasol J., Cordero T. (2010). The Production of Submicron Diameter Carbon Fibers by the Electrospinning of Lignin. Carbon.

[250] Suen D.W.-S., Chan E.M.-H., Lau Y.-Y., Lee R.H.-P., Tsang P.W.-K., Ouyang S., Tsang C.-W. (2023). Sustainable Textile Raw Materials: Review on Bioprocessing of Textile Waste via Electrospinning. Sustainability.

[251] Refate A., Mohamed Y., Mohamed M., Sobhy M., Samhy K., Khaled O., Eidaroos K., Batikh H., El-Kashif E., El-Khatib S. (2023). Influence of Electrospinning Parameters on Biopolymers Nanofibers, with Emphasis on Cellulose & Chitosan. Heliyon.

[252] Dallmeyer I., Lin L.T., Li Y., Ko F., Kadla J.F. (2014). Preparation and Characterization of Interconnected, Kraft Lignin-Based Carbon Fibrous Materials by Electrospinning. Macromol. Mater. Eng..

[253] Wang Y., Chen L. (2012). Electrospinning of Prolamin Proteins in Acetic Acid: The Effects of Protein Conformation and Aggregation in Solution. Macromol. Mater. Eng..

[254] Liu L., Xu W., Ding Y., Agarwal S., Greiner A., Duan G. (2020). A Review of Smart Electrospun Fibers toward Textiles. Compos. Commun..

[255] Deitzel J.M., Kleinmeyer J., Harris D., Beck Tan N.C. (2001). The Effect of Processing Variables on the Morphology of Electrospun Nanofibers and Textiles. Polymers.

[256] Zhang M., Ogale A.A. (2014). Carbon Fibers from Dry-Spinning of Acetylated Softwood Kraft Lignin. Carbon.

[257] Ota A., Beyer R., Hageroth U., Müller A., Tomasic P., Hermanutz F., Buchmeiser M.R. (2021). Chitin/Cellulose Blend Fibers Prepared by Wet and Dry-Wet Spinning. Polym. Adv. Technol..

[258] Hauru L.K.J., Hummel M., Michud A., Sixta H. (2014). Dry Jet-Wet Spinning of Strong Cellulose Filaments from Ionic Liquid Solution. Cellulose.

[259] Yoo M.K., Reza M.S., Kim I.M., Kim K.J. (2015). Physical Properties and Fibrillation Tendency of Regenerated Cellulose Fiber Dry Jet-Wet Spun from High-Molecular Weight Cotton Linter Pulp/NMMO Solution. Fibers Polym..

[260] Cai J., Kimura S., Wada M., Kuga S., Zhang L. (2008). Cellulose Aerogels from Aqueous Alkali Hydroxide–Urea Solution. ChemSusChem.

[261] Wilkes A.G. (2001). The Viscose Process. Regenerated Cellulose Fibres.

[262] Kreze T., Malej S. (2003). Structural Characteristics of New and Conventional Regenerated Cellulosic Fibers. Text. Res. J..

[263] Michud A., Tanttu M., Asaadi S., Ma Y., Netti E., Kääriainen P., Persson A., Berntsson A., Hummel M., Sixta H. (2016). Ioncell-F: Ionic Liquid-Based Cellulosic Textile Fibers as an Alternative to Viscose and Lyocell. Text. Res. J..

[264] Jiang G., Huang W., Li L., Wang X., Pang F., Zhang Y., Wang H. (2012). Structure and Properties of Regenerated Cellulose Fibers from Different Technology Processes. Carbohydr. Polym..

[265] Zhang S., Chen C., Duan C., Hu H., Li H., Li J., Liu Y., Ma X., Stavik J., Ni Y. (2018). Regenerated Cellulose by the Lyocell Process, a Brief Review of the Process and Properties. Bioresources.

[266] Pocienė R., Žemaitaitienė R., Vitkauskas A. (2004). Mechanical Properties and a Physical-Chemical Analysis of Acetate Yarns. Mater. Sci..

[267] Klemm D., Heublein B., Fink H., Bohn A. (2005). Cellulose: Fascinating Biopolymer and Sustainable Raw Material. Angew. Chem. Int. Ed..

[268] Teng Y., Yu G., Fu Y., Yin C. (2018). The Preparation and Study of Regenerated Cellulose Fibers by Cellulose Carbamate Pathway. Int. J. Biol. Macromol..

[269] Erdal N.B., Hakkarainen M. (2022). Degradation of Cellulose Derivatives in Laboratory, Man-Made, and Natural Environments. Biomacromolecules.

[270] Muhammed N., Govindan N. (2022). Chemical Modification of Cotton Cellulose by Carbamation with Urea and Its Dyeability with Reactive Dyes without the Use of Electrolyte. J. Nat. Fibers.

[271] Fu F., Yang Q., Zhou J., Hu H., Jia B., Zhang L. (2014). Structure and Properties of Regenerated Cellulose Filaments Prepared from Cellulose Carbamate–NaOH/ZnO Aqueous Solution. ACS Sustain. Chem. Eng..

[272] Gan S., Zakaria S., Syed Jaafar S.N. (2017). Enhanced Mechanical Properties of Hydrothermal Carbamated Cellulose Nanocomposite Film Reinforced with Graphene Oxide. Carbohydr. Polym..

[273] Paunonen S., Kamppuri T., Katajainen L., Hohenthal C., Heikkilä P., Harlin A. (2019). Environmental Impact of Cellulose Carbamate Fibers from Chemically Recycled Cotton. J. Clean. Prod..

[274] Hong W., Li Q., Di Y., Sun J., Jiao T., Xu M., Zhao Z., Xing G. (2013). Preliminary Study on the Preparation of Bamboo Cellulose Carbamate. J. Nanosci. Nanotechnol..

[275] Zhu K., Qiu C., Lu A., Luo L., Guo J., Cong H., Chen F., Liu X., Zhang X., Wang H. (2018). Mechanically Strong Multifilament Fibers Spun from Cellulose Solution via Inducing Formation of Nanofibers. ACS Sustain. Chem. Eng..

[276] Qiu C., Zhu K., Zhou X., Luo L., Zeng J., Huang R., Lu A., Liu X., Chen F., Zhang L. (2018). Influences of Coagulation Conditions on the Structure and Properties of Regenerated Cellulose Filaments via Wet-Spinning in LiOH/Urea Solvent. ACS Sustain. Chem. Eng..

[277] Qi H., Cai J., Zhang L., Nishiyama Y., Rattaz A. (2008). Influence of Finishing Oil on Structure and Properties of Multi-Filament Fibers from Cellulose Dope in NaOH/Urea Aqueous Solution. Cellulose.

[278] Cai J., Zhang L., Zhou J., Qi H., Chen H., Kondo T., Chen X., Chu B. (2007). Multifilament Fibers Based on Dissolution of Cellulose in NaOH/Urea Aqueous Solution: Structure and Properties. Adv. Mater..

[279] Fu F., Zhou J., Zhou X., Zhang L., Li D., Kondo T. (2014). Green Method for Production of Cellulose Multifilament from Cellulose Carbamate on a Pilot Scale. ACS Sustain. Chem. Eng..

[280] Fink H.-P., Weigel P., Purz H.J., Ganster J. (2001). Structure Formation of Regenerated Cellulose Materials from NMMO-Solutions. Prog. Polym. Sci..

[281] Woodings C.R. (1995). The Development of Advanced Cellulosic Fibres. Int. J. Biol. Macromol..

[282] Jia B., Yu L., Fu F., Li L., Zhou J., Zhang L. (2014). Preparation of Helical Fibers from Cellulose–Cuprammonium Solution Based on Liquid Rope Coiling. RSC Adv..

[283] Kamide K., Nishiyama K. (2001). Cuprammonium Processes. Regenerated Cellulose Fibres.

[284] Hong Y.-K., Chung K.-H., Lee W.-S. (1998). Structure of Regenerated Cellulose Fibers from DMAc/LiCl Solution. Text. Res. J..

[285] Dawsey T.R., McCormick C.L. (1990). The Lithium Chloride/Dimethylacetamide Solvent for Cellulose: A Literature Review. J. Macromol. Sci. Part C.

[286] White P., Woodings C. (2001). Lyocell: The Production Process and Market Development. Regenerated Cellulose Fibres.

[287] Sayyed A.J., Mohite L.V., Deshmukh N.A., Pinjari D.V. (2019). Structural Characterization of Cellulose Pulp in Aqueous NMMO Solution under the Process Conditions of Lyocell Slurry. Carbohydr. Polym..

[288] Gavillon R., Budtova T. (2007). Kinetics of Cellulose Regeneration from Cellulose–NaOH–Water Gels and Comparison with Cellulose-*N.*-Methylmorpholine-*N.*-Oxide–Water Solutions. Biomacromolecules.

[289] Medronho B., Lindman B. (2014). Competing Forces during Cellulose Dissolution: From Solvents to Mechanisms. Curr. Opin. Colloid. Interface Sci..

[290] Sixta H., Michud A., Hauru L., Asaadi S., Ma Y., King A.W.T., Kilpeläinen I., Hummel M. (2015). Ioncell-F: A High-Strength Regenerated Cellulose Fibre. Nord. Pulp Pap. Res. J..

[291] Orange Fiber. https://orangefiber.it/process/.

[292] Li J., Tian X., Hua T., Fu J., Koo M., Chan W., Poon T. (2021). Chitosan Natural Polymer Material for Improving Antibacterial Properties of Textiles. ACS Appl. Bio Mater..

[293] Favatela M.F., Otarola J., Ayala-Peña V.B., Dolcini G., Perez S., Torres Nicolini A., Alvarez V.A., Lassalle V.L. (2022). Development and Characterization of Antimicrobial Textiles from Chitosan-Based Compounds: Possible Biomaterials Against SARS-CoV-2 Viruses. J. Inorg. Organomet. Polym. Mater..

[294] Kaurin T., Pušić T., Čurlin M. (2022). Biopolymer Textile Structure of Chitosan with Polyester. Polymers.

[295] Afzal A., Azam F., Ahmad S., Khaliq Z., Shahzad A., Qadir M.B., Hai A.M. (2021). Development and Characterization of Biodegradable Starch-Based Fibre by Wet Extrusion. Cellulose.

[296] Temesgen S., Rennert M., Tesfaye T., Nase M. (2021). Review on Spinning of Biopolymer Fibers from Starch. Polymers.

[297] Hibbert R. (2015). What Textile Fibres Are Applicable for the Layering System for the Active Ageing?. Textile-Led Design for the Active Ageing Population.

[298] De la Harpe K.M., Marimuthu T., Kondiah P.P.D., Kumar P., Ubanako P., Choonara Y.E. (2022). Synthesis of a Novel Monofilament Bioabsorbable Suture for Biomedical Applications. J. Biomed. Mater. Res. B Appl. Biomater..

[299] Mukherjee A., Kabutare Y.H., Ghosh P. (2020). Dual Crosslinked Keratin-Alginate Fibers Formed via Ionic Complexation of Amide Networks with Improved Toughness for Assembling into Braids. Polym. Test..

[300] Amjadi S., Almasi H., Ghorbani M., Ramazani S. (2020). Preparation and Characterization of TiO_2_NPs and Betanin Loaded Zein/Sodium Alginate Nanofibers. Food Packag. Shelf Life.

[301] Raza Z.A., Abid S., Banat I.M. (2018). Polyhydroxyalkanoates: Characteristics, Production, Recent Developments and Applications. Int. Biodeterior. Biodegrad..

[302] Pakalapati H., Chang C.-K., Show P.L., Arumugasamy S.K., Lan J.C.-W. (2018). Development of Polyhydroxyalkanoates Production from Waste Feedstocks and Applications. J. Biosci. Bioeng..

[303] Taib N.-A.A.B., Rahman M.R., Huda D., Kuok K.K., Hamdan S., Bakri M.K.B., Julaihi M.R.M.B., Khan A. (2023). A Review on Poly Lactic Acid (PLA) as a Biodegradable Polymer. Polym. Bull..

[304] Li Y., Wang S., Qian S., Liu Z., Weng Y., Zhang Y. (2024). Depolymerization and Re/Upcycling of Biodegradable PLA Plastics. ACS Omega.

[305] Auerbach George H., Stenton M., Kapsali V., Blackburn R.S., Houghton J.A. (2022). Referencing Historical Practices and Emergent Technologies in the Future Development of Sustainable Textiles: A Case Study Exploring “Ardil”, a UK-Based Regenerated Protein Fibre. Sustainability.

[306] QMILK. https://www.qmilkfiber.eu/?lang=en.

[307] UMORFIL^®^ Beauty Fiber^®^. https://www.umorfil.com/product.html.

[308] Babysoy. https://babysoyusa.com/pages/about-azlon-from-soy-protein-fiber.

[309] Chi F., Chen H. (2010). Fabrication and Characterization of Zein/Viscose Textibe Fibers. J. Appl. Polym. Sci..

[310] Yang J., Wang Y., Luo J., Chen L. (2018). Facile Preparation of Self-Standing Hierarchical Porous Nitrogen-Doped Carbon Fibers for Supercapacitors from Plant Protein–Lignin Electrospun Fibers. ACS Omega.

[311] Xu W., Yang Y. (2009). Drug Sorption onto and Release from Soy Protein Fibers. J. Mater. Sci. Mater. Med..

[312] Zahra H., Selinger J., Sawada D., Ogawa Y., Orelma H., Ma Y., Kumagai S., Yoshioka T., Hummel M. (2022). Evaluation of Keratin–Cellulose Blend Fibers as Precursors for Carbon Fibers. ACS Sustain. Chem. Eng..

[313] Xu X., Wang Z., Li M., Su Y., Zhang Q., Zhang S., Hu J. (2023). Reconstructed Hierarchically Structured Keratin Fibers with Shape-Memory Features Based on Reversible Secondary-Structure Transformation. Adv. Mater..

[314] Wang Y., Li P., Xiang P., Lu J., Yuan J., Shen J. (2016). Electrospun Polyurethane/Keratin/AgNP Biocomposite Mats for Biocompatible and Antibacterial Wound Dressings. J. Mater. Chem. B.

[315] Yuan J., Xing Z.-C., Park S.-W., Geng J., Kang I.-K., Yuan J., Shen J., Meng W., Shim K.-J., Han I.-S. (2009). Fabrication of PHBV/Keratin Composite Nanofibrous Mats for Biomedical Applications. Macromol. Res..

[316] Ribeiro N., Sousa S.R., van Blitterswijk C.A., Moroni L., Monteiro F.J. (2014). A Biocomposite of Collagen Nanofibers and Nanohydroxyapatite for Bone Regeneration. Biofabrication.

[317] Ekaputra A.K., Prestwich G.D., Cool S.M., Hutmacher D.W. (2011). The Three-Dimensional Vascularization of Growth Factor-Releasing Hybrid Scaffold of Poly (ɛ-Caprolactone)/Collagen Fibers and Hyaluronic Acid Hydrogel. Biomaterials.

[318] Guzmán-Soria A., Moreno-Serna V., Canales D.A., García-Herrera C., Zapata P.A., Orihuela P.A. (2023). Effect of Electrospun PLGA/Collagen Scaffolds on Cell Adhesion, Viability, and Collagen Release: Potential Applications in Tissue Engineering. Polymers.

[319] Agheb M., Dinari M., Rafienia M., Salehi H. (2017). Novel Electrospun Nanofibers of Modified Gelatin-Tyrosine in Cartilage Tissue Engineering. Mater. Sci. Eng. C.

[320] Zhang Q., Song M., Xu Y., Wang W., Wang Z., Zhang L. (2021). Bio-Based Polyesters: Recent Progress and Future Prospects. Prog. Polym. Sci..

[321] Câmara J.S., Perestrelo R., Ferreira R., Berenguer C.V., Pereira J.A.M., Castilho P.C. (2024). Plant-Derived Terpenoids: A Plethora of Bioactive Compounds with Several Health Functions and Industrial Applications—A Comprehensive Overview. Molecules.

[322] Firdaus M., Montero de Espinosa L., Meier M.A.R. (2011). Terpene-Based Renewable Monomers and Polymers via Thiol–Ene Additions. Macromolecules.

[323] Nsengiyumva O., Miller S.A. (2019). Synthesis, Characterization, and Water-Degradation of Biorenewable Polyesters Derived from Natural Camphoric Acid. Green Chem..

[324] Zeng C., Seino H., Ren J., Hatanaka K., Yoshie N. (2013). Bio-Based Furan Polymers with Self-Healing Ability. Macromolecules.

[325] Avantium. https://avantium.com/.

[326] Ma J., Pang Y., Wang M., Xu J., Ma H., Nie X. (2012). The Copolymerization Reactivity of Diols with 2,5-Furandicarboxylic Acid for Furan-Based Copolyester Materials. J. Mater. Chem..

[327] Ryu Y.S., Oh K.W., Kim S.H. (2019). Furan-Based Self-Healing Breathable Elastomer Coating on Polylactide Fabric. Text. Res. J..

[328] Sousa A.F., Vilela C., Fonseca A.C., Matos M., Freire C.S.R., Gruter G.-J.M., Coelho J.F.J., Silvestre A.J.D. (2015). Biobased Polyesters and Other Polymers from 2,5-Furandicarboxylic Acid: A Tribute to Furan Excellency. Polym. Chem..

[329] Lee J.H., Park S.H., Kim S.H. (2020). Fabrication of Bio-Based Polyurethane Nanofibers Incorporated with a Triclosan/Cyclodextrin Complex for Antibacterial Applications. RSC Adv..

[330] Choi K.K., Park S.H., Oh K.W., Kim S.H. (2015). Effect of Castor Oil/Polycaprolactone Hybrid Polyols on the Properties of Biopolyurethane. Macromol. Res..

[331] Wang X.C., Zheng Q., Yang G.S. (2007). Influence of Preparation Methods on Structure and Properties of PA6/PA66 Blends: A Comparison of Melt-mixing and in Situ Blending. J. Polym. Sci. B Polym. Phys..

[332] Brehmer B., Boom R.M., Sanders J. (2009). Maximum Fossil Fuel Feedstock Replacement Potential of Petrochemicals via Biorefineries. Chem. Eng. Res. Des..

[333] Yang H., Wentao L. (2023). Bio-based Polyamide 56: Recent Advances in Basic and Applied Research. Polym. Eng. Sci..

[334] Mutlu H., Meier M.A.R. (2010). Castor Oil as a Renewable Resource for the Chemical Industry. Eur. J. Lipid Sci. Technol..

[335] Ogunniyi D.S. (2006). Castor Oil: A Vital Industrial Raw Material. Bioresour. Technol..

[336] Xue C., Hsu K.-M., Chiu C.-Y., Chang Y.-K., Ng I.-S. (2021). Fabrication of Bio-Based Polyamide 56 and Antibacterial Nanofiber Membrane from Cadaverine. Chemosphere.

[337] Luo K., Liu J., Abbay K., Mei Y., Guo X., Song Y., Guan Q., You Z. (2023). The Relationships between the Structure and Properties of PA56 and PA66 and Their Fibers. Polymers.

[338] Bio-Based Rilsan^®^ PA11. https://www.arkema.com/global/en/products/product-finder/product/technicalpolymers/rilsan-family-products/rilsan-pa11/.

[339] Radilon^®^. https://www.radicigroup.com/en/products/plastics/pa6-pa66-pa6-10-pa6-12-radilon.

[340] Bio-Based Yarns-EVO Fulgar^®^. https://www.fulgar.com/en/feature/39/bio-based-yarns-evo#:~:text=What%20is%20EVO%C2%AE%20made%20of?%20The%20biomass%20from%20which%20EVO%C2%AE.

[341] Bio-Based Dyneema^®^ Fiber. https://www.dyneema.com/sustainability/bio-based-dyneema-fiber.

[342] Sai H., Wang M., Miao C., Song Q., Wang Y., Fu R., Wang Y., Ma L., Hao Y. (2021). Robust Silica-Bacterial Cellulose Composite Aerogel Fibers for Thermal Insulation Textile. Gels.

[343] Reimer M., Van Opdenbosch D., Zollfrank C. (2021). Fabrication of Cellulose-Based Biopolymer Optical Fibers and Their Theoretical Attenuation Limit. Biomacromolecules.

[344] Kowalczyk M., Piorkowska E., Kulpinski P., Pracella M. (2011). Mechanical and Thermal Properties of PLA Composites with Cellulose Nanofibers and Standard Size Fibers. Compos. Part A Appl. Sci. Manuf..

[345] Tang C., Wu M., Wu Y., Liu H. (2011). Effects of Fiber Surface Chemistry and Size on the Structure and Properties of Poly(Vinyl Alcohol) Composite Films Reinforced with Electrospun Fibers. Compos. Part A Appl. Sci. Manuf..

[346] Chen G., Liu H. (2008). Electrospun Cellulose Nanofiber Reinforced Soybean Protein Isolate Composite Film. J. Appl. Polym. Sci..

[347] Liao H., Wu Y., Wu M., Liu H. (2011). Effects of Fiber Surface Chemistry and Roughness on Interfacial Structures of Electrospun Fiber Reinforced Epoxy Composite Films. Polym. Compos..

[348] Liao H., Wu Y., Wu M., Zhan X., Liu H. (2012). Aligned Electrospun Cellulose Fibers Reinforced Epoxy Resin Composite Films with High Visible Light Transmittance. Cellulose.

